# Therapeutic activity of lipoxin A_4_ in TiO_2_-induced arthritis in mice: NF-κB and Nrf2 in synovial fluid leukocytes and neuronal TRPV1 mechanisms

**DOI:** 10.3389/fimmu.2023.949407

**Published:** 2023-06-14

**Authors:** Telma Saraiva-Santos, Tiago H. Zaninelli, Marília F. Manchope, Ketlem C. Andrade, Camila R. Ferraz, Mariana M. Bertozzi, Nayara A. Artero, Anelise Franciosi, Stephanie Badaro-Garcia, Larissa Staurengo-Ferrari, Sergio M. Borghi, Graziela S. Ceravolo, Avacir Casanova Andrello, Janaína Menezes Zanoveli, Michael S. Rogers, Rubia Casagrande, Felipe A. Pinho-Ribeiro, Waldiceu A. Verri

**Affiliations:** ^1^ Laboratory of Pain, Inflammation, Neuropathy, and Cancer, Department of Pathology, Londrina State University, Londrina, Paraná, Brazil; ^2^ Division of Dermatology, Department of Medicine, Washington University School of Medicine, St. Louis, MO, United States; ^3^ Vascular Biology Program, Department of Surgery, Boston Children's Hospital-Harvard Medical School, Boston, MA, United States; ^4^ Center for Research in Health Sciences, University of Northern Paraná, Londrina, Paraná, Brazil; ^5^ Department of Physiological Sciences, Center for Biological Sciences, Londrina State University, Londrina, Paraná, Brazil; ^6^ Department of Physics, Londrina State University, Londrina, Paraná, Brazil; ^7^ Department of Pharmacology, Biological Sciences Sector, Federal University of Parana, Curitiba, Parana, Brazil; ^8^ Department of Pharmaceutical Sciences, Centre of Health Sciences, Londrina State University, Londrina, Paraná, Brazil

**Keywords:** lipoxin A4, TiO2, ALX/FPR2, inflammation, TRPV1, ROS

## Abstract

**Background:**

Lipoxin A4 (LXA_4_) has anti-inflammatory and pro-resolutive roles in inflammation. We evaluated the effects and mechanisms of action of LXA4 in titanium dioxide (TiO_2_) arthritis, a model of prosthesis-induced joint inflammation and pain.

**Methods:**

Mice were stimulated with TiO_2_ (3mg) in the knee joint followed by LXA_4_ (0.1, 1, or 10ng/animal) or vehicle (ethanol 3.2% in saline) administration. Pain-like behavior, inflammation, and dosages were performed to assess the effects of LXA_4_ *in vivo*.

**Results:**

LXA_4_ reduced mechanical and thermal hyperalgesia, histopathological damage, edema, and recruitment of leukocytes without liver, kidney, or stomach toxicity. LXA_4_ reduced leukocyte migration and modulated cytokine production. These effects were explained by reduced nuclear factor kappa B (NFκB) activation in recruited macrophages. LXA_4_ improved antioxidant parameters [reduced glutathione (GSH) and 2,2-azino-bis 3-ethylbenzothiazoline-6-sulfonate (ABTS) levels, nuclear factor erythroid 2-related factor 2 (Nrf2) mRNA and Nrf2 protein expression], reducing reactive oxygen species (ROS) fluorescent detection induced by TiO2 in synovial fluid leukocytes. We observed an increase of lipoxin receptor (ALX/FPR2) in transient receptor potential cation channel subfamily V member 1 (TRPV1)^+^ DRG nociceptive neurons upon TiO_2_ inflammation. LXA_4_ reduced TiO_2_‐induced TRPV1 mRNA expression and protein detection, as well TRPV1 co-staining with p-NFκB, indicating reduction of neuronal activation. LXA_4_ down-modulated neuronal activation and response to capsaicin (a TRPV1 agonist) and AITC [a transient receptor potential ankyrin 1 (TRPA1) agonist] of DRG neurons.

**Conclusion:**

LXA_4_ might target recruited leukocytes and primary afferent nociceptive neurons to exert analgesic and anti-inflammatory activities in a model resembling what is observed in patients with prosthesis inflammation.

## 1 Introduction

Total joint replacement recovers joint function, reduces pain, and improves quality of life (1–4). Total knee arthroplasty is a common procedure for joint replacement, which is expected to increase in the coming years (5, 6). In Europe, 2.5 million knee arthroplasties were recorded from 1975 to 2018 (7), and by the year 2030, 3.5 million procedures are expected in the United States (8). Despite the success of arthroplasty, deterioration of prosthetic components is the most associated complication. This event is characterized by the release of metallic nanoparticles that promote osteolysis, necessitating arthroplasty revision (8–10). Titanium is widely used in the production of orthopedic prostheses (11). However, TiO_2_ is the main trigger in prosthesis wear process-induced arthritis. Resident macrophages are activated and release tumor necrosis factor-alpha (TNF-α) and interleukin-1 beta (IL-1β) upon TiO_2_ phagocytosis (12). Intra-articular (i.a.) administration of TiO_2_ induces chronic arthritis and phenocopies the articular inflammation and pain caused by the release of prosthesis components upon wear (13). The available therapies for prosthesis-induced arthritis patients include non-steroidal anti-inflammatory drugs (NSAIDs), corticosteroids, and opioids. These drugs promote tolerance, but are accompanied by adverse effects or addiction, affecting life quality and economic cost (14, 15). Therefore, investigating novel candidates for prosthesis-induced arthritis treatment is crucial. If a novel therapy presents different side effects, it might benefit patients whose need are not well-served by the current treatments due to side effects.

In fact, pain is a debilitating symptom of arthritis with consequences not only in the productivity, but also in the lifestyle and social interactions of patients. Pain is also one of the major reasons that patients seek medical care (16, 17). In peripheral inflammatory pain, the initiating factor of inflammation can, for instance, activate tissue-resident cells such as macrophages, which will produce inflammatory molecules, including cytokines and reactive oxygen species (ROS) (18). Cytokines such as TNF-α, IL-1β, and IL-6 as well as ROS such as superoxide anions have a role in recruiting leukocytes, thus changing the cellular profile in the inflammatory foci (19, 20). Not only do these mediators activate nuclear factor kappa B (NF-κB); NF-κB also induces their production (21). Cytokines and ROS can also sensitize the primary nociceptor sensory neurons causing hyperalgesia (18, 22). Nociceptor neuron sensitization can involve both enhancement of the function and increased production of ion channels that facilitate neuronal firing (23). Transient receptor potential (TRP) channels such as TRPV1 and TRPA1 are examples of ion channels expressed in the axons and cell bodies of primary afferent nociceptor neurons. TRP channels have been studied as targets for novel analgesics in cancer and neuropathic and chronic pain (24, 25).

Lipoxin A4 (LXA_4_) is a specialized pro-resolving lipid mediator (SPM) derived from arachidonic acid (26). This endogenous molecule plays anti-inflammatory and resolutive roles in inflammation (27, 28). LXA_4_ acts in the nanogram range, diminishing cell recruitment, chemotaxis, and polymorphonuclear cell adhesion, thus controlling inflammatory tissue damage (29). For example, in an acute liver failure model, LXA_4_ reduces pro-inflammatory cytokine levels and inhibits apoptosis (30). In addition, LXA_4_ reduces inflammatory pain by suppressing mechanical and thermal hyperalgesia (31, 32). LXA_4_ potently blocks ROS action via nuclear factor erythroid 2-related factor 2 (Nrf2)-dependent mechanisms in several animal models (33–37). LXA_4_ also reduces NF-κB activity, accounting for an essential anti-inflammatory mechanism (30, 38–40). LXA_4_ acts through G protein coupled receptors (GPCR) for LXA_4_ (ALXR), also known as FPRL1 and FPR2 (41–43). The activation of ALX/FPR2 receptor explains most of the anti-inflammatory, pro-resolving, and protective actions of LXA_4_ (29, 42, 44). The multiple sites of actions and cellular mechanisms demonstrate that LXA_4_ has relevant properties for therapeutic development (29). Some of the LXA_4_ mechanisms are relevant in the disease development in TiO_2_ articular inflammation such as oxidative stress and cytokine production (13). Therefore, we reason that LXA_4_ merits investigation of its anti-inflammatory and analgesic activities in the context of prosthesis wearing process-released components like TiO_2_, which we pursued in the present study.

## 2 Materials and methods

**Figure 1:**
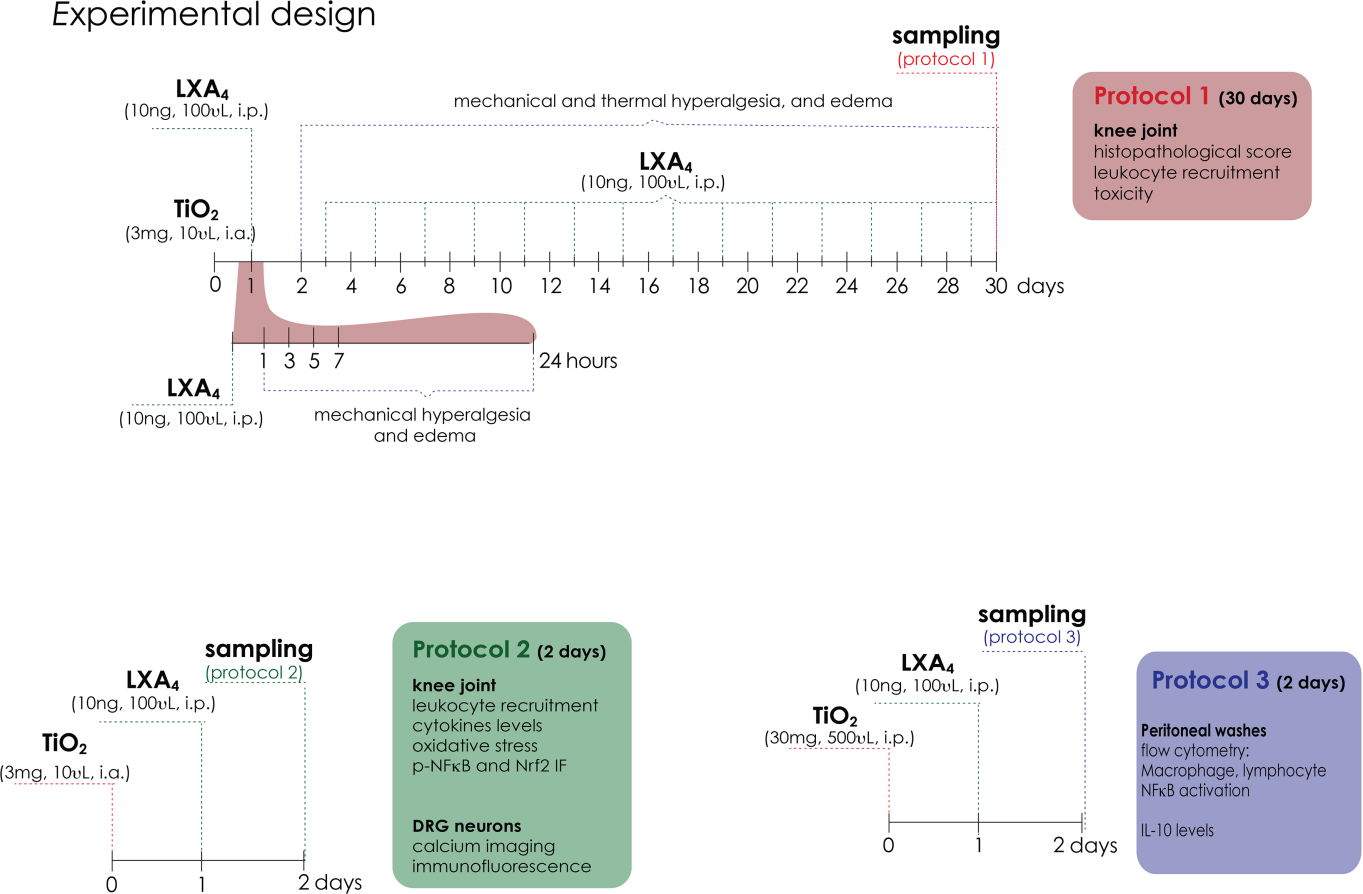
Experimental design. Protocol 1 is a 30-day experimental design. Mice were treated for 30 days with LXA_4_ (0.1, 1, and 10 ng/animal, i.p.) or vehicle (ethanol) starting 24 h after i.a. injection of TiO_2_ (3 mg/joint). Mechanical hyperalgesia and edema were evaluated 1, 3, 5, 7, 24 h (day 1), and subsequently every 2 days until the 30th day. Thermal hyperalgesia was evaluated on day 1 and every 3 days until day 30. On day 30, the knee joint was collected for histopathological analysis and toxicity assays, and the knee joint wash was collected for leukocyte recruitment. Protocol 2 is a 2-day experimental design. Mice were treated with a single treatment of LXA_4_ (10 ng/animal) starting 24 h after i.a. injection of TiO_2_ (3 mg/joint). On the 2nd day, knee joint wash was collected for leukocyte recruitment, knee joint cytokine levels, NF-κB phosphorylation, and oxidative stress (GSH, ABTS, ROS assay, and Nrf2 expression and activation). On the 2nd day of the model, DRG samples (L4–L6) were collected for calcium imaging (TRPV1 and TRPA1 agonists) and dissected for immunofluorescence (ALX/FPR2 receptor co-stained with TRPV1; TRPV1 and p-NF-κB co-staining with TRPV1 and TRPA1) and RT-qPCR. Protocol 3 is TiO_2_-induced peritonitis. Mice were given an i.p. injection of TiO_2_ (30 mg/500 µl), and after 24 h, the animals were treated with LXA_4_ (10 ng/animal) or vehicle (saline) (100 µl per animal, i.p.). Peritoneal washes were collected on the 2nd day to count total recruited leukocytes, for differential cell counts in stained slices, and for flow cytometry of lymphocytes (CD45^+^ CD4^+^), macrophages (CD45^+^ F4/80^+^), and p-NF-κB.

For detailed materials and methods, please refer to the **Supplementary Data 1**. Briefly, **Figure 1** shows the experimental protocols in which male Swiss (20–25 g) received the administration of intra-articular TiO_2_ (3 mg/10 µl/knee joint) as previously described (13) to induce aseptic arthritis. The first experiments were dedicated to determining the disease phenotype upon LXA_4_ treatment (Fig ure 1; protocol 1); for this, we performed the treatment with LXA_4_ (0.1, 1, or 10 ng) or vehicle (3.2% ethanol/saline) [100 µl per animal, intraperitoneal (i.p.)] 24 h after TiO_2_ stimulus. Mechanical hyperalgesia was assessed using an electronic esthesiometer (45) in different time points for 30 days to perform a dose–response curve. The most effective dose of LXA_4_ (10 ng/animal, every 48 h) was used for succeeding experiments. Edema was evaluated using measurements of the transverse diameters using a caliper and thermal hyperalgesia by Hargreaves apparatus. Knee joint lavages were collected on the 30th day to assess the total and differential leukocyte recruitment (46). Stomach was collected to assess myeloperoxidase (MPO) activity (gastric damage) (47) and blood samples were used to assess serum levels of aspartate transaminase (AST), alanine transaminase (ALT) (liver damage), urea, and creatinine (renal damage) (48). Hematoxylin–eosin (HE) staining was performed on knee joint samples for histopathology analysis (49).

Inflammation and pain were present by the 2nd day of TiO_2_ arthritis and LXA_4_ activity could be observed. Chronic alterations were already studied with the experimental approach described in the previous paragraph. Considering these points, we reasoned that mechanistic studies could be performed on the 2nd day of TiO_2_ arthritis to reduce the duration of inflammation to which the animals were exposed. Therefore, potential mechanisms of LXA_4_ were studied in the early stages of TiO_2_-induced pain and inflammation (**Figure 1**; protocol 2). To this end, we collected the knee joint in the 2nd day after stimulus injection to determine leukocyte recruitment, and to assess the cytokine levels by enzyme-linked immunosorbent assay (ELISA) (TNF-α, IL-1β, IL-6, and IL-10 levels). Oxidative stress was measured by reduced glutathione (GSH), 2,2-azino-bis(3-ethylbenzothiazoline-6-sulfonate) (ABTS) measurement (50–52), and Nrf2 mRNA expression by reverse transcriptase-quantitative real-time polymerase chain reaction (RT-qPCR). Synovial fluid leukocytes were collected for p-NFκB and Nrf2 staining by immunofluorescence (53), and total ROS was measured using the probe 2’,7’-dichlorofluorescein diacetate (DCF-DA). Ipsilateral dorsal root ganglia (DRG) (corresponding to L4–L6 segments) were also dissected 2 days after TiO_2_ to perform calcium influx imaging using confocal microscopy (54), transient receptor potential cation channel subfamily V member 1 (TRPV1) mRNA expression by RT-qPCR, and TRPV1, TRPA1, ALX/FPR2, and p-NFκB staining by immunofluorescence.

The limited number of cells in the synovial fluid led us to use a TiO_2_-triggered peritonitis model to assess the cellular profile of recruited leukocytes and NF-κB activation in macrophages. We also determined if treatment with LXA_4_ could modulate the responses triggered by TiO_2_ via flow cytometry (**Figure 1**; protocol 3). For this approach, mice received an i.p. injection of TiO_2_ (30 mg/500 µl), and 24 h after TiO_2_ stimulus (post-treatment), mice were treated with LXA_4_ (10 ng) or vehicle (saline) (100 µl per animal, i.p.). After an additional 24 h, peritoneal washes were collected in FACS buffer (10 ml per animal), and total leukocyte recruitment was counted, and flow cytometry (54) for CD45, CD4, F4/80, and p-NFκB staining was performed. All experimental conditions were standardized by our laboratory as previously published (13, 49, 54, 55) and in preliminary experiments performed for this manuscript.

For *in vivo* experiments, we used 6, 8, or 10 mice in each group per experiment depending on the methodology (indicated in the figure legends). *In vitro* experiments with DRG samples were performed using an *n* of 4 pools (10 mice to form 1 pool) per group. Two-way ANOVA followed by Tukey’s post-test was used to compare all groups and doses when responses were measured at different times after the stimulus injection. The analyzed factors were treatments, time, and time versus treatment interaction. Parametric results were evaluated by one-way ANOVA followed by Tukey’s post-test for data from a single time point. Kruskal–Wallis followed by Dunn post-test or two-way were used for non-parametric results. *p* < 0.05 was considered significant.

## 3 Results

### 3.1 Treatment with LXA_4_ reduces TiO_2_-induced articular mechanical hyperalgesia, thermal hyperalgesia, and edema in mice

**Figure 2:**
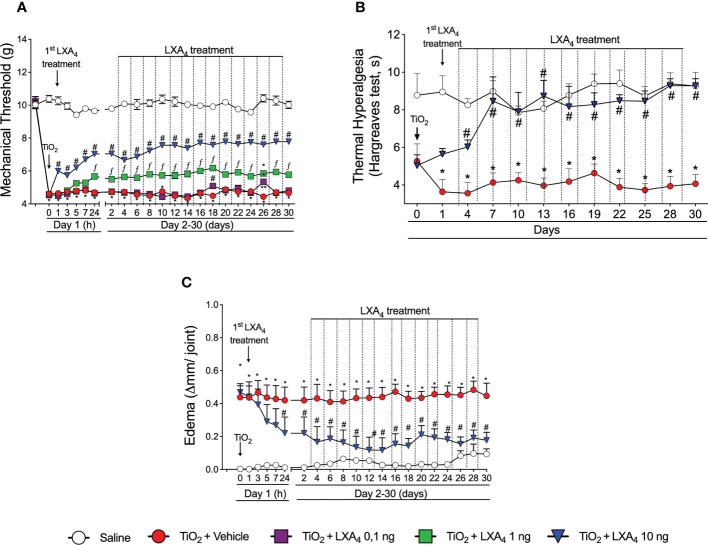
LXA_4_ inhibits TiO_2_-induced articular mechanical hyperalgesia, thermal hyperalgesia, and edema in the knee joint. Mice were treated for 30 days with LXA_4_ (0.1, 1, and 10 ng/animal, i.p.; 48-h intervals) or vehicle (ethanol) starting 24 h after i.a. injection of TiO_2_ (3 mg/joint). Mechanical hyperalgesia **(A)** was evaluated 1, 3, 5, 7, 24 h (day 1), and subsequently every 2 days until day 30. Thermal hyperalgesia **(B)** was evaluated on day 1 and every 3 days until day 30. Results are expressed as mean ± SEM, *n* = 6 mice per group per experiment and are representative of two separate experiments [*p < 0.05 vs. saline group; #*p* < 0.05 vs. TiO_2_ group; **p < 0.05 vs. TiO_2_ and LXA_4_ (10 ng) groups; *fp* < 0.05 vs. TiO_2_ and LXA_4_ (10 and 1 ng) groups, repeated measures two-way ANOVA followed by Tukey’s post-test]. Edema **(C)** was evaluated 1, 3, 5, 7, 24 h (day 1), and subsequently every 2 days until day 30.

A dose–response curve was performed to assess the potential analgesic and anti-inflammatory effects of LXA_4_ in TiO_2_-induced arthritis. Treatment started 24 h after i.a. TiO_2_ injection. We could still observe significant analgesia by the 24th hour after LXA_4_ treatment, which was reduced by the 48th hour (data not shown). Therefore, treatments with LXA_4_ were performed every 48 h. The injection of 3 mg/joint of TiO_2_ induced mechanical hyperalgesia, and treatment with LXA_4_ reduced the mechanical hyperalgesia in a dose-dependent (0.1, 1, or 10 ng/animal, 100 μl i.p.) manner. The most effective dose was 10 ng/animal, which was chosen for the subsequent experiments (**Figure 2A**). TiO_2_ also induced thermal hyperalgesia that was reduced by LXA_4_ 10 ng/animal treatment. The reduction of thermal hyperalgesia was observed from the 4th day onwards, with complete inhibition from the 7th to the 30th day (**Figure 2B**).

We also investigated if the treatment with LXA_4_ reduces knee joint edema. A dose of 10 ng/animal of LXA_4_ significantly reduced TiO_2_-induced articular edema 24 h after the first treatment, with persistent anti-inflammatory effect until the 30th day of arthritis (**Figure 2C**). The saline-injected group did not develop edema (**Figure 2C**).

### 3.2 LXA_4_ reduces TiO_2_-induced joint histopathology changes and inhibits leukocyte recruitment to the articular space

**Figure 3:**
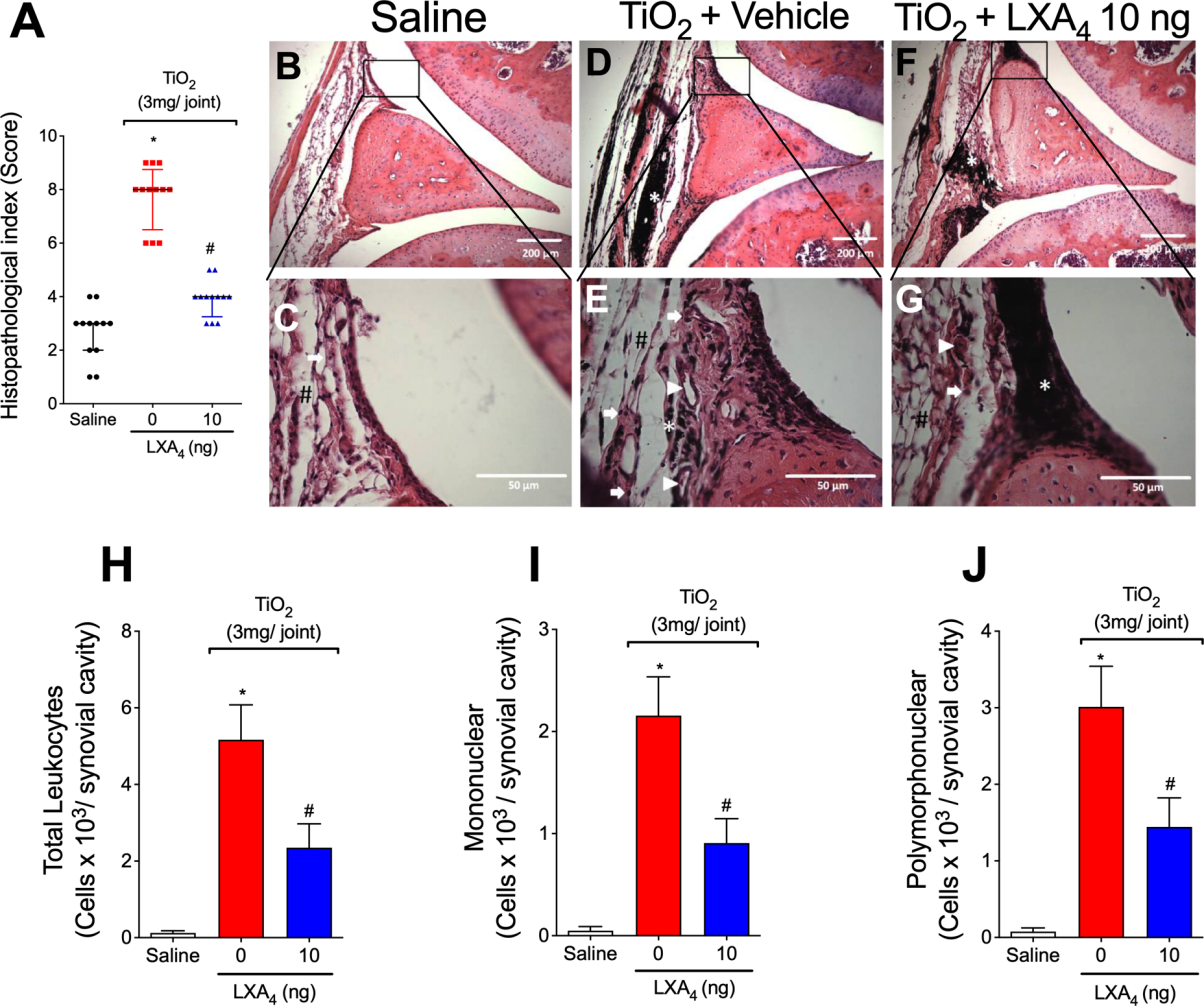
LXA_4_ reduces TiO_2_-induced histopathological damage and recruitment in the knee joint. Mice were treated with LXA_4_ (10 ng/animal, i.p.) or vehicle (ethanol) 24 h after i.a. TiO_2_ (3 mg) injection and on alternate days for 30 days. On the 30th day, the knee joints were collected and stained with HE. Histopathological index **(A)** and analysis **(B–G)**. The panel shows: saline **(B, C)**, TiO_2_-injected treated with vehicle **(D, E)**, and TiO_2_-injected treated with LXA_4_ **(F, G)**. The representative image demonstrated the invasive pannus (#), leukocyte infiltration (arrow), angiogenesis (arrowhead), and TiO_2_ nanoparticles (asterisk). Original magnification 10× **(B, D, F)** and 40× **(C, E, G)**. Results are expressed as mean ± SEM, *n* = 12 mice per group per experiment, two independent experiments (*p < 0.05 vs. saline group; #*p* < 0.05 vs. TiO_2_ group, Kruskal–Wallis followed by Dunn’s post-test). On the 30th day, knee joint washes were collected to count total leukocytes **(H)**, mononuclear **(I)**, and polymorphonuclear cells **(J)**. Results are expressed as mean ± SEM, *n* = 6 mice per group per experiment, two independent experiments (*p < 0.05 vs. saline group; #*p* < 0.05 vs. TiO_2_ group, one-way ANOVA followed by Tukey’s post-test).

Mice were treated with LXA_4_ (10 ng/animal, i.p., every 48h) or vehicle (3.2% ethanol in saline) 24 h after i.a. TiO_2_ (3 mg) injection. On the 30th day, the knee joint was collected for HE histopathology evaluation (**Figures 3A–G**). LXA_4_ reduced TiO_2_-induced synovial hyperplasia, inflammatory infiltrates, and vascular proliferation observed in the histopathological index analyses (**Figure 3A**). Treatment with vehicle showed no effect on TiO_2_-induced histopathological changes.

Leukocyte recruitment to the knee joint is a hallmark of arthritis (56). To investigate the effect of LXA_4_ on leukocyte recruitment 30 days post-TiO_2_ stimulus, knee joint washes were collected to evaluate the total number of leukocytes and mononuclear and polymorphonuclear cells. The injection of TiO_2_ significantly increases the number of leukocytes recruited to the knee joint 30 days after the stimulus (**Figures 3H–J**). Our results show that the treatment with LXA_4_ at 10 ng/animal reduced TiO_2_-induced recruitment of total leukocyte (**Figure 3H**) and mononuclear (**Figure 3I**) and polymorphonuclear cells (**Figure 3J**).

### 3.3 LXA_4_ does not induce liver, kidney, or stomach damage

**Figure 4:**
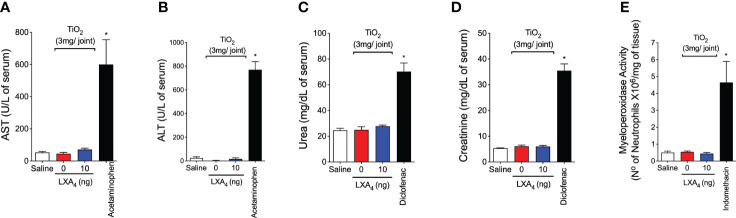
LXA_4_ chronic treatment does not induce toxicity. Mice were treated for 30 days with LXA_4_ (10 ng/animal, i.p. q.o.d.) starting 24 h after i.a. injection of TiO_2_ (3 mg/joint), and serum and stomach were collected. AST **(A)**, ALT **(B)**, urea **(C)**, and creatinine **(D)** serum levels and MPO activity in the stomach **(E)** were determined to evaluate treatment toxicity. As positive drug control for gastric, hepatic, and renal toxicity, indomethacin (2.5 mg/kg, i.p., diluted in tris/HCl buffer, for 7 days), acetaminophen (650 mg/kg, i.p., diluted in saline), and diclofenac (200 mg/kg, p.o., diluted in saline) were used, respectively. Results are expressed as mean ± SEM, *n* = 6 mice per group per experiment, two independent experiments (*p < 0.05 vs. all groups, one-way ANOVA followed by Tukey’s post-test).

Thirty days after TiO_2_ stimulus, serum samples and stomach were collected to evaluate whether the chronic treatment with LXA_4_ would induce gastric, hepatic, or renal damage, which are common side effects of non-steroidal anti-inflammatory drugs (56). Toxicity was assessed through the concentrations of AST, ALT, urea, and creatinine, and MPO activity (**Figure 4**). The treatment with 10 ng/animal qod of LXA_4_ did not modify the serum concentration of AST, ALT (**Figures 4A, B**), urea, creatinine (**Figures 4C, D**), or MPO activity in the stomach compared with positive controls (**Figure 4E**). Therefore, our data suggest that chronic treatment does not induce detectable gastric, hepatic, or renal lesion/damage.

### 3.4 LXA_4_ reduces TiO_2_-induced leukocyte recruitment, cytokines production, and NF-κB activation in macrophages

In the following experiments, we opted to reduce the treatment period to investigate the inflammatory and pain mechanisms of LXA_4_. We considered that **Figures 2**–**4** established the beneficial effect of LXA_4_ treatment during a chronic period and that inflammation and pain achieved significant development by the second day of arthritis. This approach allowed us to reduce the suffering of animals and investigate the mechanisms involved in LXA_4_ post-treatment of ongoing TiO_2_ arthritis.

**Figure 5:**
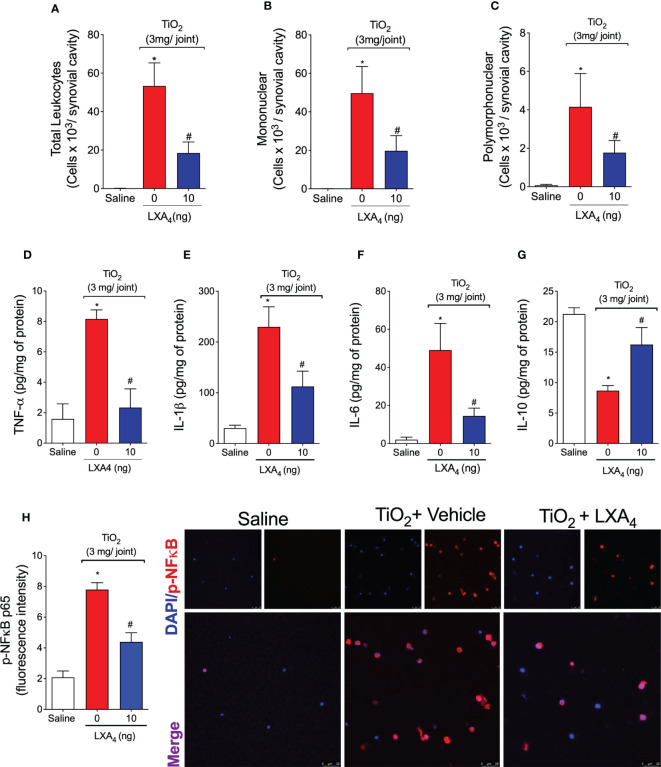
LXA_4_ modulates TiO_2_-induced leukocyte recruitment, cytokine production, and NF-κB activation. Mice received a single treatment of LXA_4_ (10 ng/animal) 24 h after i.a. injection of TiO_2_ (3 mg/joint), and on the 2nd day, knee joint washes were collected to count total leukocytes **(A)**, mononuclear **(B)**, and polymorphonuclear cells **(C)**. The knee joint was collected, and TNF-α **(D)**, IL-1β **(E)**, IL-6 **(F)**, and IL-10 **(G)** were measured by ELISA. Knee joint washes were used to perform an immunofluorescence assay. **(H)** shows the representative images of p-NF-κB p65 (red) with nuclear staining by DAPI and the quantitation. Fluorescence intensity **(H)** was analyzed by a confocal microscope at 63× magnification. Results are expressed as mean ± SEM, *n* = 6 mice per group per experiment, two independent experiments (*p < 0.05 vs. saline group; #*p* < 0.05 vs. TiO_2_ group, one-way ANOVA followed by Tukey’s post-test).

Given the role of recruited leukocytes in inflammatory pain and oxidative burst (57), we next assessed the efficacy of LXA_4_ in modulating TiO_2_-induced leukocyte recruitment after a single treatment. In this case, recruitment was evaluated on the 2nd day (**Figures 5A–C**) to further support that this time point is adequate and mimics all inflammatory features of TiO_2_ arthritis together with the pain and edema observed in **Figure 2**. The injection of TiO_2_ significantly increased the number of total leukocytes recruited on the 2nd day after the stimulus (**Figures 5A–C**). Our results show that the treatment with LXA_4_ at 10 ng/animal reduced TiO_2_-induced recruitment of total leukocyte (**Figure 5A**) and mononuclear (**Figure 5B**) and polymorphonuclear cells (**Figure 5C**). These data show that most leukocytes recruited to the joint were mononuclear cells (90%). Compared with the 30th day data (**Figures 5A–C**), 10.6-fold more leukocytes migrated in the knee joint on the 2nd day, indicating that this time point is suitable for investigating inflammatory mechanisms. Indeed, on the 2nd day, higher mononuclear cells than neutrophil counts were already established. The pathophysiological mechanisms underlying this unusual cellular profile deserves further investigation in future studies.

The potential of LXA_4_ to modulate pro-inflammatory cytokine (TNF-α, IL-1β, and IL-6) and anti-inflammatory cytokine (IL-10) production in the joint tissue was evaluated on the 2nd day (**Figures 5D–G**). The i.a. injection of TiO_2_ induced a significant increase in TNF-α (**Figure 5D**), IL-1β (**Figure 5E**), and IL-6 (**Figure 5F**). A single treatment with LXA_4_ was enough to reduce the levels of these pro-inflammatory cytokines induced by TiO_2_ (**Figures 5D–F**). Thus, the effect of LXA_4_ in reducing the production of essential cytokines represents one of its mechanisms to reduce pain, edema, and recruitment of leukocytes (58). In addition, IL-10 production was increased by LXA_4_ (**Figure 5G**), evidencing this lipid mediator’s anti-inflammatory and immunoregulatory capacity with a single treatment.

Synovial fluid leukocytes were collected on the 2nd day, and the phosphorylated (p) form of NF-κB was determined by immunofluorescence assay (**Figure 5H**). Treatment with LXA_4_ reduced the fluorescence intensity of the p-NFκB p65 subunit induced by TiO_2_ (**Figure 5H**). Therefore, these data suggest that inhibiting NF-κB activation is, at least, one of the mechanisms by which LXA_4_ ameliorates TiO_2_-induced inflammation and pain. This underscores the importance of this transcription factor to cytokine production (**Figures 5D–G**) and leukocyte recruitment (**Figures 5A–C**).

**Figure 6:**
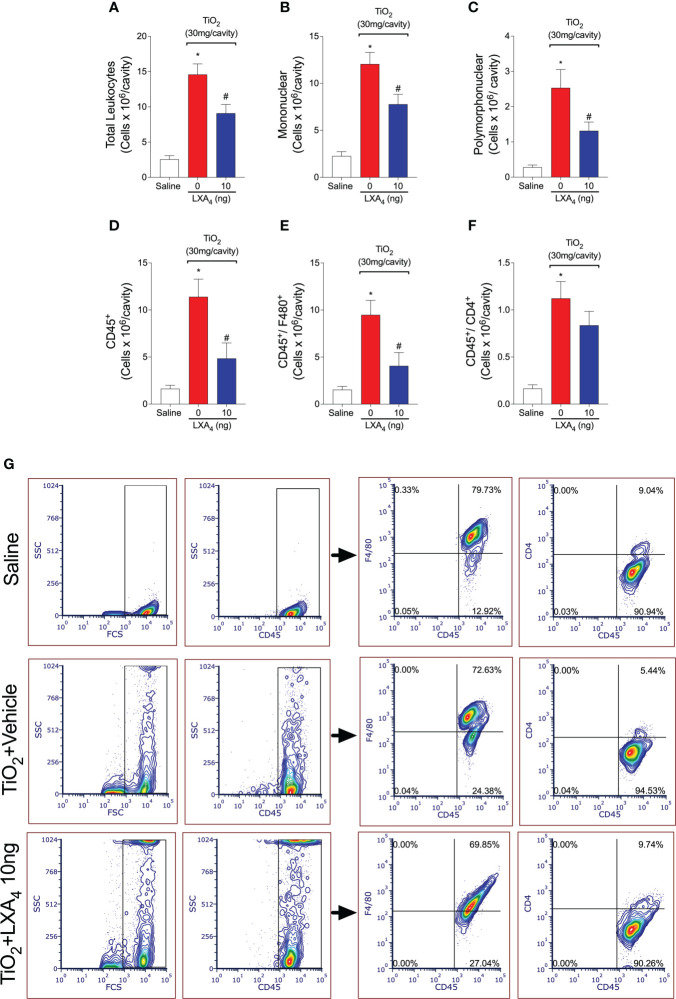
TiO_2_ increases CD45^+^ F4/80^+^ macrophages and CD45^+^ CD4 ^+^ lymphocyte counts that, in part, are down-modulated by LXA_4_. TiO_2_ peritonitis was induced with an i.p. injection of TiO_2_ (30 mg/500 µl), and after 24 h, the animals received treatment with LXA_4_ (10 ng) or vehicle (saline) (100 µl per animal, i.p.). Peritoneal washes were collected on the 2nd day to count total recruited leukocytes **(A)**, mononuclear cells **(B)**, and polymorphonuclear cells **(C)**. Flow cytometry for total leukocyte cells, CD45^+^ cells **(D)**, macrophages [CD45^+^ F4/80^+^ cells **(E)**], and lymphocytes [CD45^+^ CD4 ^+^ cells **(F)**] corrected by the total recruited leukocytes. **(G)** shows the representative gates. Results are expressed as mean ± SEM, *n* = 10 mice per group per experiment, two independent experiments (*p < 0.05 vs. saline group; #*p* < 0.05 vs. TiO_2_ group, one-way ANOVA followed by Tukey’s post-test).

The number of recovered cells in synovial washes was insufficient to perform a flow cytometry analysis in our hands. To enable further assessment of the cellular profile of leukocytes recruited upon TiO_2_ stimulation, NF-κB activation, and the effect of LXA_4_, we standardized a peritonitis model to mimic the TiO_2_-induced inflammation. The increased volume of the peritoneal cavity allows the recruitment of larger numbers of leukocytes than the knee joint. We performed a TiO_2_ dose–response (data not shown) and found that 30 mg per animal induced significant leukocyte recruitment. We performed a single post-treatment with 10 ng of LXA_4_ (similar to what was performed for the arthritis), which was sufficient to reduce the leukocyte recruitment (**Figures 6A–C**). TiO_2_ recruited mostly mononuclear cells, so we evaluated the ratio of recruited macrophages and lymphocytes, and the modulation by LXA_4_. Although we observed a similar percentage of positive cells in all analyzed groups, when we corrected the percentages by the total number of cells in the peritoneal washes, the results revealed significant differences between the groups (**Figure 6D**). We show that TiO_2_ increased the number of CD45^+^ F4/80^+^ macrophages (**Figure 6E**) and CD45^+^ CD4^+^ lymphocytes (**Figure 6F**), and treatment with LXA_4_ reduced the number of recruited CD45^+^ F4/80^+^ macrophages (**Figure 6E**), but not of CD45^+^ CD4^+^ lymphocytes (**Figure 6F**). The proportion of macrophages represents 70% of the total recruited leukocytes, and lymphocytes represent 10% of the total recruited CD45^+^ leukocyte population (**Figure 6G**). Thus, CD45^+^ F4/80^+^ macrophages represent the majority of leukocytes recruited by TiO_2_.

**Figure 7:**
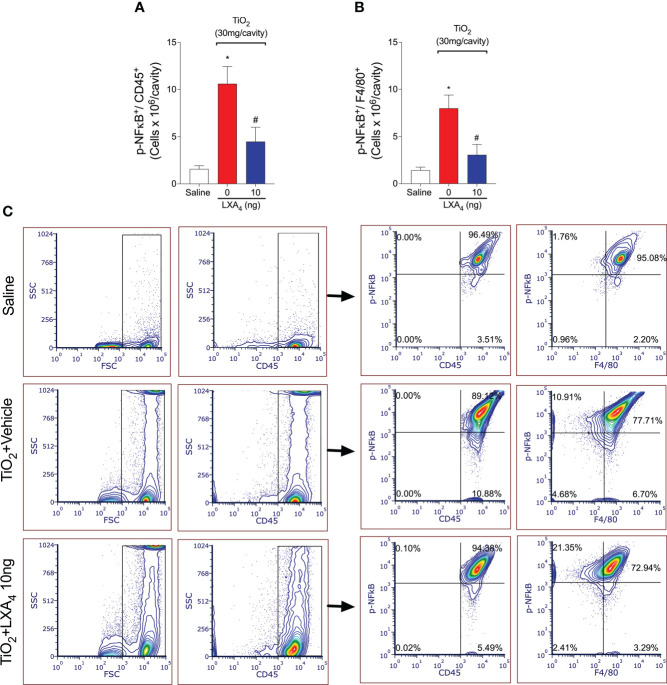
Treatment with LXA_4_ reduces TiO_2_-triggered NF-κB activation in CD45^+^ F4/80^+^ macrophages. TiO_2_ peritonitis was induced with an i.p. injection of TiO_2_ (30 mg/500 µl), and after 24 h, the animals were treated with 10 ng LXA_4_ or vehicle (saline) (100 µl per animal, i.p.). Flow cytometry for p-NFκB p65 in total leukocytes [CD45^+^ p-NFκB^+^ cells **(A)**] and macrophages [CD45^+^ F4/80^+^ p-NFκB^+^ cells **(B)**] corrected by the total number of recruited leukocytes. **(C)** shows the representative gates. Results are expressed as mean ± SEM, *n* = 10 mice per group per experiment, two independent experiments (*p < 0.05 vs. saline group; #*p* < 0.05 vs. TiO_2_ group, one-way ANOVA followed by Tukey’s post-test).

Since LXA_4_ reduced the recruitment of CD45^+^ F4/80^+^ macrophages and these are main cell population in the peritoneal cavity, we reasoned that these cells could be a cellular target of the LXA_4_-mediated reduction in activated NF-κB. To check this possibility, we performed flow cytometry and show that TiO_2_ increased NF-κB activation/phosphorylation in CD45^+^ F4/80^+^ macrophages (pNF-κB^+^ F4/80^+^ cells) (**Figure 7B**). Quite interestingly, CD45^+^ F4/80^+^ pNF-κB^+^ macrophages represent 85% of the total NF-κB^+^ CD45^+^ cells (**Figures 7A, C**), and LXA_4_ treatment reduced this activation. Although we have not exhaustively investigated the role of each cell type in TiO_2_ inflammation and LXA_4_ activity, these data show that CD45^+^ F4/80^+^ macrophages are the main mononuclear cell population in TiO_2_ inflammation and a target of LXA_4_ activity with respect to both cellular recruitment and NF-κB activation.

### 3.5 LXA_4_ inhibits oxidative stress improving antioxidant capacity in mice

**Figure 8:**
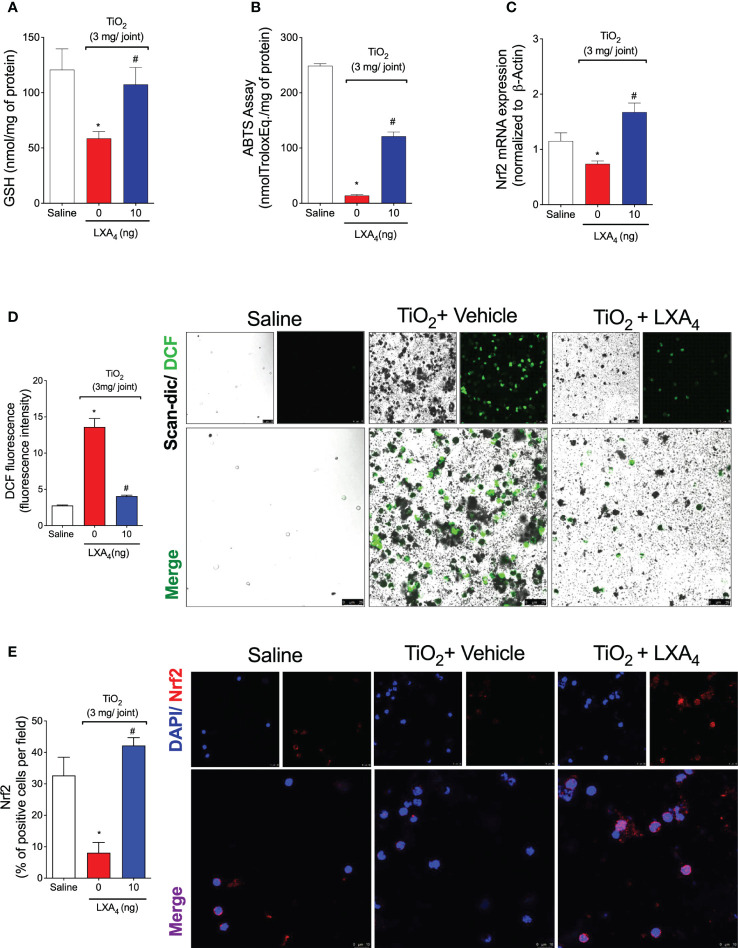
LXA_4_ inhibits TiO_2_-induced oxidative stress, improving antioxidant capacity. Mice received a single treatment of LXA_4_ (10 ng/animal) 24 h after i.a. injection of TiO_2_ (3 mg/joint), and on the 2nd day, the knee joint was collected, and the antioxidant effect of LXA_4_ was measured using GSH levels **(A)** and ABTS **(B)**. Nrf2 mRNA expression was quantitated by RT-qPCR **(C)**. Dihydrofluorescein diacetate (DCF-DA) was added to knee joint wash cells for 30 min, and intracellular ROS levels from intact cells were analyzed using the scan-dic and green channel in a confocal microscope at 63× magnification. DCF fluorescence intensity **(D)** indicates ROS production, which was quantitated. Representative images show DCF fluorescence for the negative control, TiO_2_, and LXA_4_ groups **(D)**. Knee joint washes were used to perform an immunofluorescence assay. **(E)** shows the representative images of Nrf2 (red) with nuclear staining by DAPI, and the quantitation by % of positive cells per field. The data **(E)** were analyzed by a confocal microscope at 63× magnification with 1.5× zoom. TiO_2_ nanoparticles are the black pigments. Results are expressed as mean ± SEM, *n* = 6 mice per group per experiment, two independent experiments (*p < 0.05 vs. saline group; #*p* < 0.05 vs. TiO_2_ group, one-way ANOVA followed by Tukey’s post-test).

Knee joint samples were collected on the 2nd day of TiO_2_ arthritis, and antioxidant capacity was measured with GSH and ABTS assays (**Figures 8A, B**). In other models, TiO_2_ induces the production of ROS and, consequently, oxidative stress in various organs (59–61). Herein, we show that TiO_2_ stimulus reduced the levels of endogenous antioxidants in the knee joint tissues as observed in free radical scavenging ability and GSH levels (**Figures 8A, B**). On the 2nd day, a single treatment with LXA_4_ significantly restored the levels of ABTS and GSH (**Figures 8A, B**), demonstrating that treatment with LXA_4_ reestablished the antioxidant ability to scavenge free radicals such as ABTS cationic radical and positively upregulates the endogenous antioxidant GSH. GSH is upregulated by the transcription factor Nrf2 (62), and we observed that LXA_4_ increases the Nrf2 mRNA expression (**Figure 8C**). Then, because these phenomena were observed in the knee joint tissue and recruited leukocytes have a major role in those alterations, we analyzed the recruited leukocytes.

ROS production was measured in the synovial fluid leukocytes using a DCF-DA probe, which, when oxidized, generates a fluorescence product (DCF) proportional to overall intracellular ROS levels. We observed that treatment with LXA_4_ reduced DCF fluorescence intensity (**Figure 8D**), demonstrating that treatment with LXA_4_ inhibits TiO_2_-induced production of ROS (**Figure 8D**). Articular fluids were collected on the 2nd day, and Nrf2 was determined by immunofluorescence assay (**Figure 8E**). Supporting the qPCR data, we observed that treatment with LXA_4_ increased the percentage of Nrf2-positive cells per field (**Figure 8E**).

### 3.6 TiO_2_ increases the ALX/FPR2 receptor expression on nociceptive TRPV1^+^ neurons

**Figure 9:**
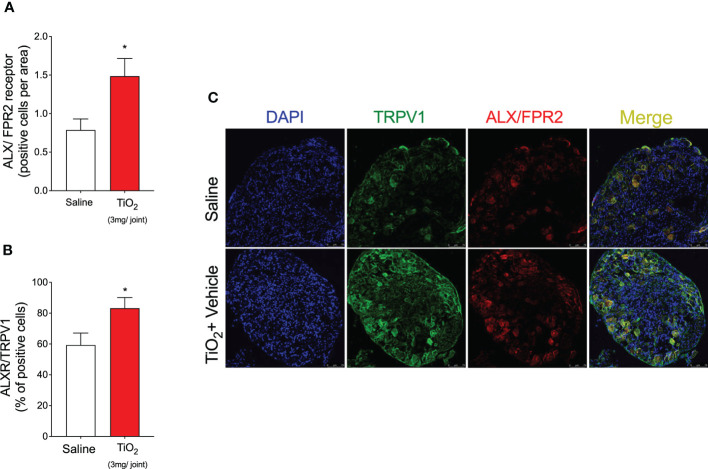
TiO_2_ increases ALX/FPR2 expression on nociceptive neurons. On the 2nd day of the model, DRGs samples (L4–L6) were dissected and immunofluorescence staining for TRPV1 and ALX/FPR2 was performed. Panels **(A, B)** show the quantitative analyses of the number of ALX/FPR2 receptor-positive cells per area **(A)** and co-staining with TRPV1 (as a percent of positive cells) **(B)**. **(C)** shows the representative images of TRPV1^+^ cells (green), ALX/FPR2 receptor-positive cells (red), and the merge of double labeling of TRPV1 and ALX/FPR2 on DRG (20× magnification with 1.0 zoom in). Results are expressed as mean ± SEM, *n* = 8 mice per group per experiment, two independent experiments (*p < 0.05 vs. saline group, Student’s *t*-test).

LXA_4_ acts through the receptor ALX/FPR2 in peripheral tissues and regulates cellular responses of interest in inflammation and resolution (29). ALX/FPR2 is expressed in tissues and cell types such as immune cells, fibroblasts, epithelial cells, and astrocytes (29, 32). The effect of LXA_4_ in reducing mechanical and thermal hyperalgesia indicates that it could, eventually, act on nociceptor neurons. To suggest a neuronal effect of LXA_4_, it was necessary to determine (1) if the nociceptor sensory neurons express ALX/FPR2 and (2) whether LXA_4_ shapes the neuronal profile and activity. These were our next steps. We investigated the expression of ALX/FPR2 receptor in the DRG by immunofluorescence staining for ALX/FPR2 receptor and TRPV1, which is a TRP channel expressed by nociceptive C-fibers (**Figure 9**). Our data show that TiO_2_ increased the expression of ALX/FPR2 receptor in the DRG of mice (**Figures 9A, C**). We also found that TiO_2_ increases the percent of double positive ALXR/TRPV1 cells, indicating nociceptor sensory neurons express ALX/FPR2 receptor, which is enhanced in this neuronal population in TiO_2_ inflammation (**Figures 9B, C**). Altogether, these results suggest that nociceptive TRPV1^+^ neurons are targets of the action of LXA_4_ during TiO_2_-induced arthritis.

### 3.7 LXA_4_ reduces TiO_2_-induced TRPV1 activation and expression on DRG neurons

**Figure 10:**
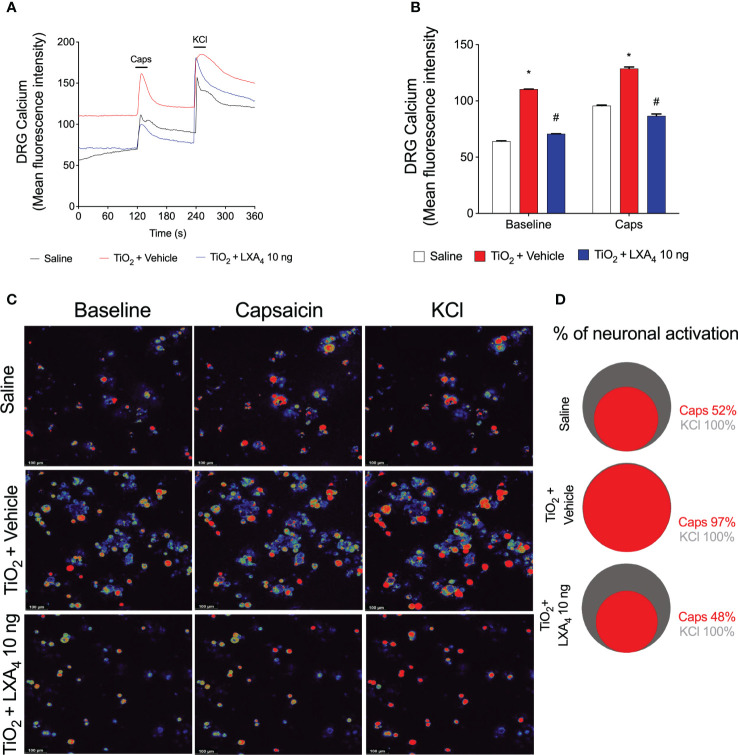
LXA_4_ reduces TiO_2_-induced TRPV1 activation on DRG neurons. Mice received a single treatment of LXA_4_ (10 ng/animal) 24 h after i.a. injection of TiO_2_ (3 mg/joint). On the 2nd day, the DRGs (L4–L6) were collected for calcium imaging using Fluo-4 AM probe. The fluorescence intensity traces of calcium-fluo-4 in representative DRG fields during the 6 min of recording is shown in panel **(A)**. **(B)** displays the mean fluorescence intensity of calcium-fluo-4 at baseline (0‐s mark) and that following the stimulus with capsaicin (a TRPV1 agonist, 120‐s mark). Response to KCl (activates all neurons) begins at the 240‐s mark. **(C)** shows representative fields of DRG neurons (baseline fluorescence, the fluorescence after capsaicin, and after KCl). **(D)** shows a Venn diagram comparing the percent of the neuronal population that is capsaicin-responsive (red) within those neurons that responded to KCl control (gray). Results are expressed as mean ± SEM, *n* = 4 DRG seeded plates (each plate is a neuronal culture pooled from 10 mice) per group per experiment, two independent experiments (*p < 0.05 vs. saline group; #*p* < 0.05 vs. TiO_2_ group, two-way ANOVA followed by Tukey’s post-test).

**Figure 11:**
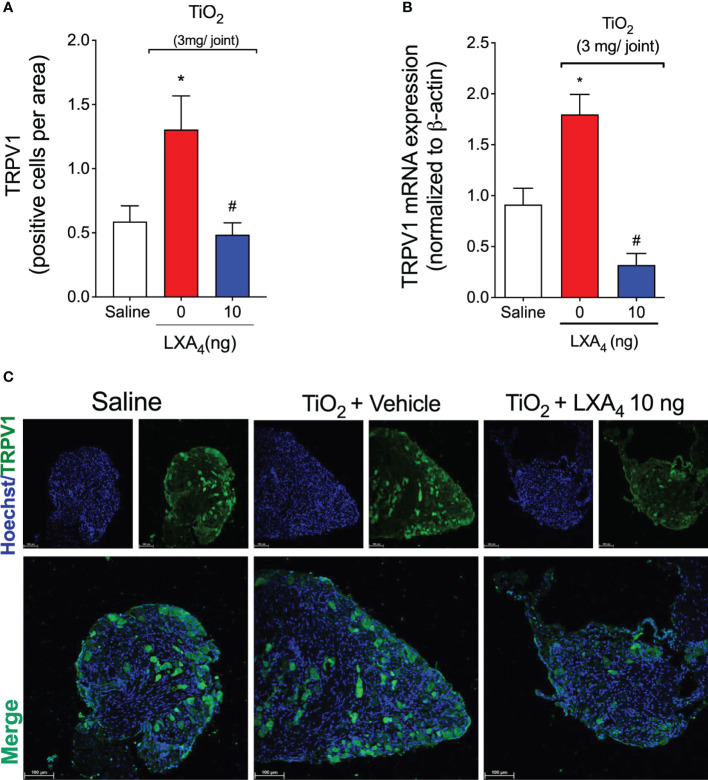
LXA_4_ inhibits TiO_2_-induced TRPV1 expression in DRG neurons. On the 2nd day of the model, DRG samples (L4–L6) were dissected for TRPV1 staining by immunofluorescence and for mRNA expression by RT-qPCR. **(A)** (quantitation) and **(C)** (representative images) show the number of positive cells per area TRPV1 (green) with nuclear staining by Hoechst 33342 in DRGs (20× magnification with 1.0 zoom in). **(B)** shows the DRG RT‐qPCR data, demonstrating that LXA_4_ reduced TiO_2_‐induced TRPV1 mRNA expression. Results are expressed as mean ± SEM, *n* = 8 mice per group per experiment, and RT‐qPCR used *n* = 6 mice per group per experiment, two independent experiments (*p < 0.05 vs. saline group; #*p* < 0.05 vs. TiO_2_ group, one-way ANOVA followed by Tukey’s post-test).

Considering the results of **Figure 9**, our next step was to assess neuronal activation. This was quantified using calcium influx as measured by a fluorescent probe Fluo-4 AM in DRG neurons (63). We investigated whether DRG neurons from TiO_2_‐stimulated mice would present an increase in the baseline calcium levels and response to capsaicin (TRPV1 agonist) stimulation compared to saline-injected controls mice, and the ability of LXA_4_ to modulate this response (**Figure 10**). DRG neurons from vehicle-treated mice presented a higher baseline level of calcium influx than saline mice or LXA_4_-treated DRGs (**Figures 10A–C**). These data suggest that LXA_4_ reduces the activation of DRG neurons in TiO_2_-induced inflammation because the increase in calcium influx is indicative of DRG neuron activity (**Figures 10A–C**). Notably, in addition to the diminished basal level of calcium, LXA_4_ treatment also reduced the responsiveness of DRG neurons to capsaicin, which is a TRPV1 agonist (**Figures 10A–C**). The treatment with LXA_4_ reduced by 49% the capsaicin-responsive neurons compared to the TiO_2_ + vehicle group as per the Venn diagram (**Figure 10D**). Corroborating with the reduction of neuronal activation and diminished response to capsaicin, we demonstrated that treatment with LXA_4_ inhibited the increase of TRPV1 (**Figure 11B**) mRNA expression induced by TiO_2_, as well as TRPV1 staining in the DRG (**Figures 11A, C**). Therefore, LXA_4_ inhibits TiO_2_-induced DRG protein detection, mRNA expression, and activity of a critical ion channel (TRPV1) to nociceptor sensory neuron sensitization (64), which resulted in a functional outcome of reduced neuronal responsiveness and pain upon LXA_4_ treatment.

**Figure 12:**
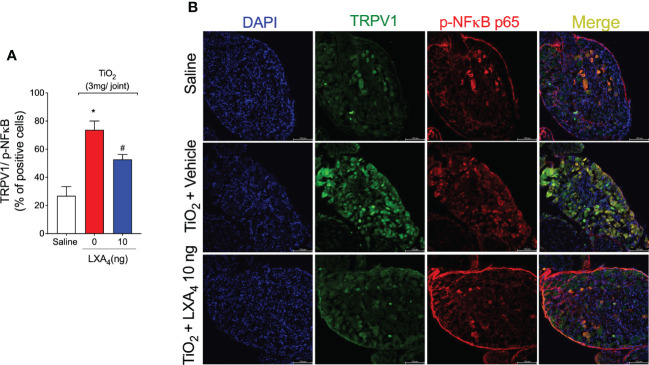
LXA_4_ reduces TiO_2_-induced NF-κB activation in TRPV1 positive neurons. On the 2nd day of the model, DRG samples (L4–L6) were dissected for immunofluorescent TRPV1 and p65 p-NF-κB staining. **(A)** shows the percent of positive cells co-stained with p65 p-NF-κB. **(B)** shows representative images of TRPV1+ cells (green), p65 p-NF-κB positive cells (red), and the merge of double labeling (TRPV1 and NF‐κB) in DRG samples (20× magnification with 1.0× zoom). DAPI was used for nuclear staining. Results are expressed as mean ± SEM, *n* = 8 mice per group per experiment, two independent experiments (*p < 0.05 vs. saline group; #*p* < 0.05 vs. TiO_2_ group, one-way ANOVA followed by Tukey’s post-test).

We also investigated whether TRPV1^+^ neurons co-expressed p-NFκB in the TiO_2_-induced DRG as a surrogate marker of neuronal activation. Immunofluorescence shows that the intra-articular injection of TiO_2_ increased the percentage of TRPV1^+^ neurons co-stained with p-NFκB, and that treatment with LXA_4_ can reduce it (**Figures 12A, B**). These data further demonstrate that TRPV1^+^ neurons are activated in TiO_2_ inflammation and that LXA_4_ treatment reduces their activation.

### 3.8 LXA_4_ reduces TiO_2_-induced TRPA1 activation on DRG neurons

TRPA1^+^ neurons in the dorsal root ganglion are involved in inflammation-induced hyperalgesia in peripheral tissues (65–68). Therefore, to further explore the neuronal mechanism involved in the model and the role of LXA_4_, we investigated whether LXA_4_ modulates TRPA1 channels in TiO_2_-induced arthritis. To achieve this aim, we investigated whether DRG neurons from stimulated mice would present increase of calcium levels in response to AITC (a TRPA1 agonist) stimulation and the modulation by LXA_4_ (**Figure S1**). We observed that LXA_4_ treatment reduced the increased responsiveness of DRG neurons to AITC induced by TiO_2_ (**Figures S1A–C**). The treatment reduced by 37% the number of responsive neurons to AITC compared to the TiO_2_ + vehicle group as per the Venn diagram (**Figure S1D**). Moreover, TRPA1 staining was enhanced in DRG neurons in the TiO_2_ group, and one treatment with LXA_4_ decreased the density of TRPA1 stained neurons (**Figures S2A, C**). Co-staining of TRPA1 with p-NFκB showed that TiO_2_ did not induce the activation of NF-κB in TRPA1^+^ nociceptive neurons (**Figures S2B, C**). These data show (**Figures 9**–**12**; **Figures S1**, **S2**) the importance of the function of TRPV1 and TRPA1 ion channels in this model of inflammatory pain and that LXA_4_ reduced the activity of both TRP ion channels. These results also show that the transcription factor regulation occurring in nociceptive neurons can be different depending on the neuronal population profile, an observation that merits further investigation.

## 4 Discussion

LXA_4_ administration reduced chronic ongoing TiO_2_-induced joint edema, mechanical and thermal hyperalgesia, leukocyte recruitment, and histopathological changes. LXA_4_ activity was explained by a reduction in pro-inflammatory cytokines (TNF‐α, IL‐1β, and IL-6) and an increase in the anti-inflammatory cytokine IL-10. Corroborating these data, LXA_4_ reduced NFκB activation in synovial fluid leukocytes. In the TiO_2_ inflammation context, we demonstrated that CD45^+^ F4/80^+^ macrophages are the main recruited leukocyte type induced by TiO_2_, and that 85% of NFκB activation occurs in these cells. LXA_4_ reduced both cell recruitment and activation of NFκB in CD45^+^ F4/80^+^ macrophages. Furthermore, a single treatment with LXA_4_ significantly restored free-radical scavenging ability (ABTS) and GSH levels. It reduced the production of ROS, accompanied by increased Nrf2 mRNA expression in the knee joint tissue and protein staining in synovial fluid leukocytes, supporting an antioxidant effect. Altogether, this demonstrates that LXA_4_ has anti-inflammatory and antioxidant effects in TiO_2_-induced arthritis. Moreover, we show that TiO_2_ injection increased the production of the LXA_4_ receptor protein, ALX/FPR2, by TRPV1^+^ neurons. In DRGs, LXA_4_ decreased TiO_2_‐induced mRNA expression and protein staining of the pain‐related ion channel TRPV1. In terms of neuronal function, LXA_4_ reduced the activation of DRG neurons, as determined by lower baseline levels of calcium influx in DRG, and reduced responsiveness to TRPV1 activation by capsaicin stimulation, and to TRPA1 activation by AITC stimulation. Furthermore, treatment with LXA_4_ did not induce gastric, hepatic, or renal damage, indicating its safety compared to common side effects of non-steroidal anti-inflammatory drugs.

Intra-articular administration of TiO_2_ induces a response that resembles prosthesis joint inflammation and pain (13). Pain is a cardinal symptom of joint inflammation and is a direct cause of the decision to seek medical care, limitation of limb function, and decreased quality of life (69). Therefore, the development of novel therapeutics that are effective for optimal pain management is critical in prosthesis wear process-induced arthritis. TiO_2_ arthritis is, in principle, an aseptic inflammation and the opposite of septic arthritis such as that induced by intraarticular injection of *Staphylococcus aureus*. Evidence demonstrates that limiting the endogenous production and action of LXA_4_ by genetic deletion of 5-lipoxygenase and antagonizing the ALX/FPR2 receptor with BOC-2, respectively, improve the immune response against *S. aureus* by avoiding the downregulation of dendritic cells’ recruitment by LXA_4_ (70). However, that context is different from that of the present study. Here, we demonstrate a beneficial effect of exogenous LXA_4_ treatment in aseptic prosthesis arthritis, while that previous study showed that endogenous LXA_4_ has a detrimental role in septic arthritis. Exemplifying aseptic inflammatory conditions, LXA_4_ levels were decreased in synovial fluid of patients with rheumatoid arthritis and osteoarthritis, suggesting that downmodulation of LXA_4_ is a permissive factor to aseptic chronic joint diseases with high and low inflammation profiles (71). These observations agree with the present findings.

Prosthesis wear process-released particles, such as TiO_2_, activate macrophages to produce various pro-inflammatory mediators, growth factors, and pro-inflammatory lipids (57, 72), and these molecules orchestrate the inflammatory response (73). LXA_4_ and other agonists of ALX/FPR2 can downregulate those inflammatory mechanisms. LXA_4_ inhibited synoviocyte proliferation and also decreased the levels of IL-6, IL-1β, and TNF-α in rheumatoid arthritis (74). Of interest, LXA_4_ downregulates TNF-α-directed neutrophil trafficking (75). The ALX/FPR2 agonist (AT-01-KG) reduced neutrophilic inflammation, CXCL1, and IL-1β production and enhanced neutrophil apoptosis in a model of gout arthritis (76). LXA_4_ diminishes pain in the non-compressive lumbar disc herniation model by inhibiting production of pro-inflammatory cytokines (TNF-α, and IL-1β) and upregulating IL-10 and transforming growth factor-beta (TGF-β) (38). Treatment with LXA_4_ also increases anti-inflammatory cytokine (TGF-β and IL-10) levels after exposition to ultraviolet light (77). IL-10 restricts the polarization of M1 macrophages, blocks the IL-33/ST2 axis during arthritis (78), and inhibits neutrophil recruitment, matrix metalloproteinases activity, edema (79), and pain (80). We show that LXA_4_ reduced TNF-α, IL-1β, and IL-6 levels and increased IL-10 levels in TiO_2_-induced arthritis. Thus, reducing pro-inflammatory cytokines and increasing anti-inflammatory cytokines, which orchestrate the inflammatory and nociceptive responses, might contribute to LXA_4_ alleviation of leukocyte recruitment, edema, and mechanical and thermal hyperalgesia. A limitation of the present study is that we did not identify the cells in which cytokine production was downregulated by LXA_4_. Macrophages and lymphocytes are important cells in the production of both pro-inflammatory (81) and anti-inflammatory (82, 83) cytokines. For instance, macrophages produce pro-inflammatory cytokines in response to TiO_2_ stimulus (12, 84). Also, M2 macrophages (85, 86) and regulatory T (Treg) cells (87, 88) produce IL-10 to limit inflammation. LXA_4_ has differential actions in M1 and M2 macrophages. LXA_4_ reduces the gene expression of pro-inflammatory cytokines in M1 macrophages and increases the IL-10 mRNA expression in M2 macrophages derived from THP-1 cells (89). LXA_4_ also induces M2 polarization in a model of osteoarthritis (90). Thus, LXA_4_ can both induce macrophage polarization towards the M2 profile and stimulate these cells to produce IL-10. The overexpression of 15-lipoxygenase in mesenchymal stem cells (MSC) can enhance LXA_4_ production, and consequently, MSC overexpressing 15-lipoxygeanse can shape the balance between Th17/Treg by increasing Treg and IL-10 production (91). Thus, macrophages and lymphocytes are potential sources of cytokines and targets of LXA_4_ activity.

Administration of TiO_2_ particles bypasses the wait for prosthesis wear and reduces the number of animals needed to investigate mechanisms and novel treatments for this condition. Chronic inflammation is responsible for peri-prosthetic osteolysis and aseptic loosening of the prosthesis (92, 93). Macrophage-like synoviocytes are resident cells in the synovium lining. They are responsible for the phagocytosis of prosthetic wear particles, production of pro-inflammatory cytokines such as IL-1β and TNF-α, triggering inflammation, recruitment of immune cells, and activation of fibroblast-like synoviocytes (94, 95). During aseptic loosening, a significant number of macrophages infiltrate into peri-implant tissues (96). In our model, total leukocytes in the synovial cavity were higher in the early stage (2nd day) than in the late stage (30th day). We also observed higher counts of mononuclear cells than neutrophils on the 2nd day post-TiO_2_ administration, which is unexpected considering the leukocyte recruitment kinetics of most inflammatory responses. We show that the CD45^+^ F4/80^+^ macrophage population is the main (up to 70%) recruited leukocyte type in the TiO_2_-inflammation and seems to be an important target of LXA_4_ anti-inflammatory effects. Further studies are necessary to investigate the underlying mechanisms for these specific leukocyte kinetics in TiO_2_ arthritis. LXA_4_ reduced inflammatory cytokine production induced by TiO_2_, which lined up well with the reduced p-NFκB staining in synovial fluid leukocytes. NF-κB exerts its transcription factor activity and regulates the expression of various genes encoding pro-inflammatory cytokines, which have been shown to play essential roles in inflammation. Diminished NF-κB activation reduces the production of pro-inflammatory cytokines and downmodulates inflammatory reactions (21). Our findings corroborate prior evidence that LXA_4_ inhibits NF-κB in other disease models (97–99). Furthermore, LXA_4_ suppresses the LPS-induced proliferation of RAW264.7 macrophages by targeting the NF-κB pathway (100). The treatment with LXA_4_ reduced LPS-evoked TNF-α production and inhibited NF-κB activation in a coculture system using RAW264.7 cells and human colon carcinoma cell line (Caco-2) (101). We observed that TiO_2_ induced NF-κB activation mostly in CD45^+^ F4/80^+^ macrophages (85% of the total CD45+ pNF-κB+ cells). LXA_4_ treatment reduced the NF-κB activation in CD45^+^ F4/80^+^ macrophages triggered by TiO_2_.

Oxidative stress has an essential role in inflammatory pain (102). Reactive oxygen and nitrogen species (ROS and RNS, respectively) produced during inflammation contribute directly to nociceptor neuron activation (103). TiO_2_ induces lipid peroxidation, DNA damage, and protein breakdown, corroborating the presence of oxidative stress (104). LXA_4_ increases antioxidant capacity via Nrf2 in various models (33, 37, 77, 105). Herein, we demonstrated the *in vivo* antioxidant effect of LXA_4_ and induction of Nrf2, explaining the mechanism of protection against oxidative stress by increasing endogenous antioxidants as per GSH and ABTS assays. GSH is a downstream target of Nrf2 activity (106), and our data on GSH together with the literature (33, 77) guided the choice of investigating Nrf2. In arthritis, synovial fluid cells are crucial in the production of ROS, which can increase the level of NF-κB-dependent pro-inflammatory cytokines and promote the formation of an amplification loop that feeds back to further elevation of additional ROS (107). Prosthesis wear particles can induce oxidative stress in macrophage culture (108). On the other hand, LXA_4_ treatment increases nuclear translocation of Nrf2 in cardiomyocytes (109). In cultured cortical astrocytes exposed to oxygen-glucose deprivation/recovery insults, LXA_4_ reduced oxidative stress by enhancing the Nrf2 pathway (37). We show that LXA_4_ inhibits TiO_2_-triggered ROS generation and enhances Nrf2 in synovial fluid leukocytes. Altogether, these data indicate that LXA_4_ enhances Nrf2 expression, and reduces cytokine, ROS production, and, importantly, TiO_2_-triggered NF-κB activation. The modulation of p-NFκB and Nrf2 by LXA_4_ may also involve their competition to bind to CREB (cAMP-responsive element-binding protein) (62).

LXA_4_ has an analgesic effect in various conditions, ranging from acute inflammation (31, 32) to neuropathic pain (110). Our data show that LXA_4_ has an analgesic effect on ongoing prothesis-wearing-like chronic arthritis (30 days) at 10 ng/animal. LXA_4_ reduced mechanical and thermal hyperalgesia and provided 2 days of analgesia per treatment. Other SPMs such as maresin (MaR) MaR1 and MaR2 and resolvin D1 and D2 reduce inflammatory pain by inhibiting the expression and/or activity of DRG neurons’ TRPV1 and TRPA1 (54, 111–113). Thus, some SPMs can modulate ion channels to induce analgesia, suggesting that this mechanism should also be investigated for LXA_4_ in TiO_2_-induced arthritis. However, to that end, we first needed to ascertain if nociceptive neurons express the ALX/FPR2 receptor in TiO_2_ arthritis. We observed that ALX/FPR2 receptor staining was increased in TiO_2_-induced arthritis, and more specifically, TRPV1^+^ nociceptive neurons express ALX/FPR2 receptor and that TiO_2_ inflammation enhances the percentage of ALXR^+^/TRPV1^+^ neurons. Thus, DRG TRPV1^+^ neurons are likely more susceptible to LXA_4_ action during TiO_2_ arthritis than when uninflamed, supporting the analgesic effect of LXA_4_. A single post-treatment with LXA_4_ reduced ongoing DRG neuronal activation (baseline calcium levels) and prevented capsaicin-induced TRPV1 activation of DRG neurons. Explaining the diminished neuronal activation by LXA_4_, this SPM reduced TiO_2_‐induced TRPV1 mRNA expression and protein staining (and co-stained with p-NFκB p65) in DRG neurons. To our knowledge, this is the first work to demonstrate that LXA_4_ reduces TRPV1 channel mRNA expression and protein staining in DRG neurons. Notably, this resulted in diminished TRPV1 activity, causing analgesia. TRPV1 is expressed by approximately 54% of DRG neurons, and TRPA1 is expressed by approximately 22% of DRG neurons. Most TRPA1 channels are co-expressed with TRPV1 in DRG neurons (114) and there is evidence that they can also dimerize as a mechanism of nociceptor sensitization (115). Those TRPA1 channels that are not co-expressed with TRPV1 represent a sub-population of neurons involved in neuropathic pain and not in inflammatory pain (115). Corroborating the literature about the role of TRPA1 in pain and its interaction with TRPV1 (67, 114, 115), as well as the present results on TRPV1, we also observed that TiO_2_ enhances the neuronal activation by a TRPA1 agonist and TRPA1 staining, both of which were inhibited by LXA_4_. However, in contrast to what was observed in TRPV1^+^ neurons, TiO_2_ did not induce an increase of p-NFκB in TRPA1^+^ neurons, indicating that the role of the transcription factor NF-κB is likely not the same in all DRG nociceptor neuron populations. Thus, the mechanism of action of LXA_4_ depends, at least in part, on down-modulating the activity of TRP channels essential to nociceptor neuronal sensitization and chronic pain (116). Our study also contributes to building the concept that targeting ion channels is part of the mechanisms of action of SPM.

**Figure 13:**
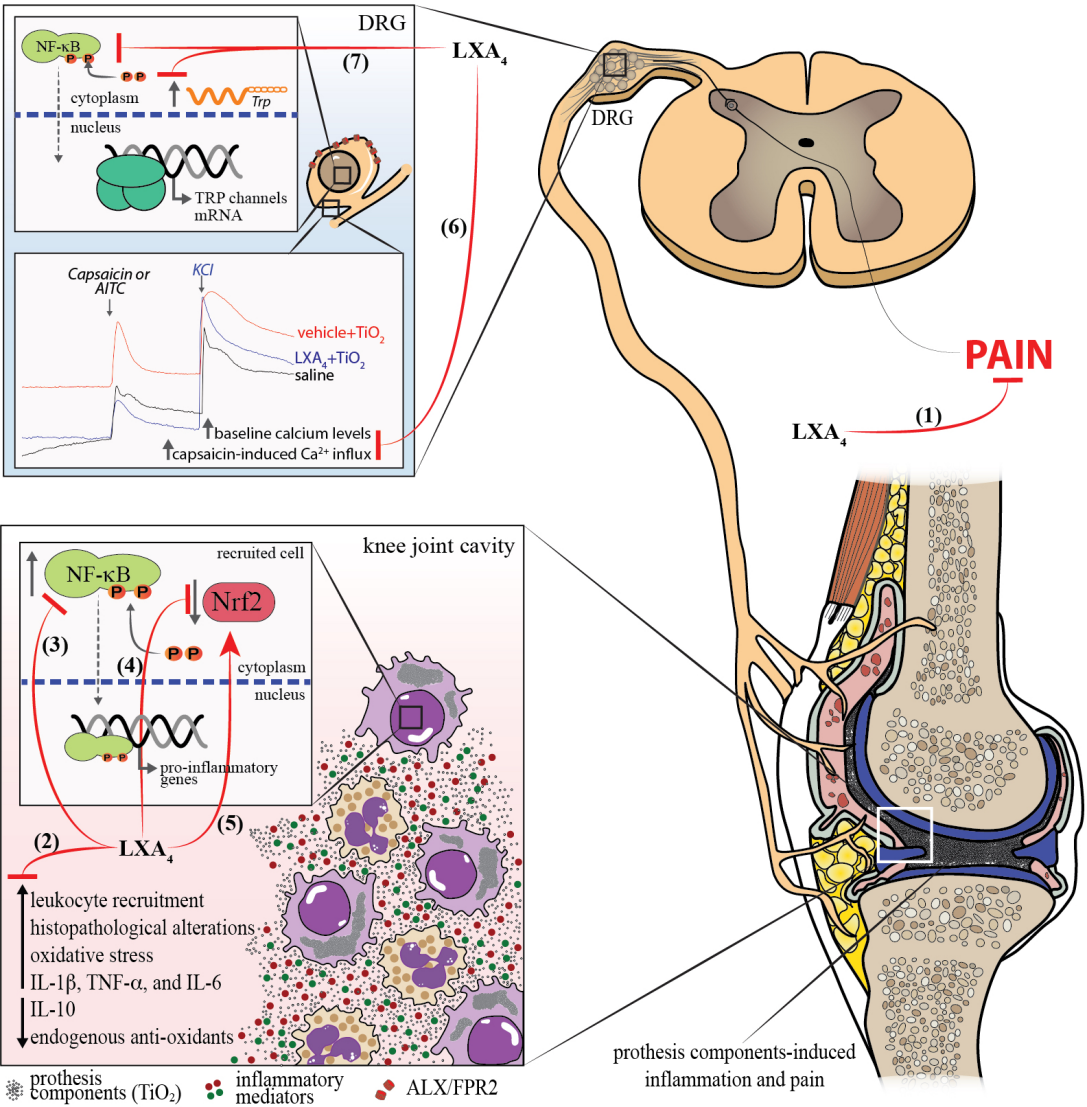
Mechanism of action of LXA_4_ in TiO_2_-induced arthritis. (1) LXA_4_ treatment reduces chronic articular pain induced by TiO_2_. (2) LXA_4_ reduces leukocyte recruitment to the knee joint at early and late TiO_2_-induced arthritis stages, histopathological alterations, oxidative stress, and IL-1β, TNF-α, and IL-6 production, and increases production of endogenous antioxidants and IL-10. These anti-inflammatory findings were supported by the (3) decreased NF-κB activation in macrophages. (4, 5) LXA_4_ increases the Nrf2 mRNA expression and activation, which were reduced by TiO_2_. (6) We also demonstrated that LXA_4_ reduces the activation of DRG neurons in TiO_2_ inflammation by decreasing the baseline neuronal activation and capsaicin/AITC-induced calcium influx (7) and increasing TRPV1 mRNA expression and protein staining (and co-stained with p-NFκB^+^), and TRPA1 staining induced by TiO_2_. TRPV1^+^ nociceptive neurons express ALX/FPR2 receptor, and TiO_2_ inflammation enhances the ALXR^+^/TRPV1^+^ neurons. Finally, all these mechanisms explain the analgesic (1) and anti-inflammatory (2) effects of LXA_4_ in this animal model of prosthesis-wearing-process-released components (e.g., TiO_2_)-induced arthritis.

We demonstrated that LXA_4_ has therapeutic effects against ongoing chronic TiO_2_ arthritis, favorably altering knee joint pathology. **Figure 13** is a schematic representation of the mechanism of action of LXA_4_ in TiO_2_-induced arthritis. TiO_2_ triggered the production of cytokines and ROS to induce inflammation and pain. The activation of NF-κB and down-modulation of Nrf2 are mechanisms occurring, at least, in synovial fluid leukocytes that amplify inflammatory cytokines and oxidative stress pathways in response to TiO_2_. LXA_4_ targets these pathways. LXA_4_ could reduce recruitment and NF-κB activation mainly in CD45^+^ F4/80^+^ macrophages. We further observed that LXA_4_ attenuated the staining of the nociceptor-neuron-sensitization-related ion channels TRPV1 and TRPA1, unveiling a hitherto unknown nociceptor neuron mechanism of LXA_4_. To sum up, this study demonstrated that LXA_4_ is a promising approach to treating complications related to prosthesis-induced inflammation and pain by inhibiting the activation of synovial fluid leukocytes and primary afferent nociceptor sensory neurons.

## Data availability statement

The raw data supporting the conclusions of this article will be made available by the authors, without undue reservation.

## Ethics statement

The animal study was reviewed and approved by Londrina State University Ethics Committee on Animal Research and Welfare (process number 11147.2016.40).

## Author contributions

Performed experiments: TS-S, THZ, MFM, KCA, CRF, MMB, NAA, AF, SB-G, LS-F, SMB. Methodological support: GSC, ACA, JMZ, MSR, RC, FAP-R, WAV. Data analysis: TS-S, THZ, MFM, KCA, CRF, MMB, NAA, AF, SB-G, LS-F, SMB, GSC, ACA, JMZ, MSR, RC, FAP-R, WAV. Supervision: RC, FAP-R, WAV. Funding: GSC, ACA, JMZ, MSR, RC, FAP-R, WAV. Conceived the study: RC, FAP-R, WAV. Wrote the first draft: TS-S, WAV. Editing and approval of final version: All authors.
