# Supplementary material for: Therapeutic activity of lipoxin A4 in TiO2-induced arthritis in mice: NF-κB and Nrf2 in synovial fluid leukocytes and neuronal TRPV1 mechanisms
Source: Front Immunol. 2023 Jun 14;14:949407. doi: 10.3389/fimmu.2023.949407 (PMC10304281; doi:10.3389/fimmu.2023.949407)
Supplement: Supplementary file 1 [file DataSheet_1.docx]

Supplementary Material

Therapeutic activity of Lipoxin A_4_ in TiO_2_-induced arthritis in mice: NF-κB and Nrf2 in synovial fluid leukocytes and neuronal TRPV1 mechanisms

Telma Saraiva-Santos^1,2^, Tiago H. Zaninelli^1,2,3^, Marília F. Manchope^1^, Ketlem C. Andrade^1^, Camila R. Ferraz^1^, Mariana M. Bertozzi^1^, Nayara A. Artero^1^, Anelise Franciosi^1^, Stephanie Badaro-Garcia^1^, Larissa Staurengo-Ferrari^1^, Sergio M. Borghi^1,4^, Graziela S. Ceravolo^5^, Avacir Casanova Andrello^6^, Janaína Menezes Zanoveli^7^, Michael S. Rogers^3^, Rubia Casagrande^8^, Felipe A. Pinho-Ribeiro^2*^ and Waldiceu A. Verri Jr^1*^

^1^Laboratory of Pain, Inflammation, Neuropathy, and Cancer, Department of Pathology, Londrina State University, Londrina, Paraná, Brazil.

^2^ Division of Dermatology, Department of Medicine, Washington University School of Medicine, St. Louis, MO 63110, USA.

^3^ Vascular Biology Program, Department of Surgery, Boston Children's Hospital-Harvard Medical School, Boston, Massachusetts, USA.

^4^ Center for Research in Health Sciences, University of Northern Paraná, Londrina, Paraná, Brazil.

^5^ Department of Physiological Sciences, Center for Biological Sciences, Londrina State University, Londrina, Paraná, Brazil.

^6^ Department of Physics, Londrina State University, Londrina, Paraná, Brazil.

^7^ Department of Pharmacology, Biological Sciences Sector, Federal University of Parana, Curitiba, Parana, Brazil.

^8^ Department of Pharmaceutical Sciences, Centre of Health Sciences, Londrina State University, Londrina, Paraná, Brazil.

*** Correspondence:**Waldiceu A. Verri Jr
[waverri@uel.br](mailto:waverri@uel.br)

Felipe A. Pinho-Ribeiro

[dfelipe@wustl.edu](mailto:dfelipe@wustl.edu)

1. **Supplementary Data 1**

## Animals

Male Swiss (20-25 g) mice were used. Mice were housed in standard clear plastic cages with free access to water and food, a light/dark cycle of 12/12h, and a controlled temperature (21°±1°C). Animals were acclimated to the testing room at least one hour before the experiments, and all behavioral testing was performed between 9 a.m. and 5 p.m. Animal care and handling procedures were developed accordingly to the International Association for Study of Pain (IASP) guidelines and with the approval of the Londrina State University Ethics Committee on Animal Research and Welfare (process number 11147.2016.40). All efforts were made to minimize the number of animals used and their suffering.

## Experimental procedures

The experiments dedicated to determining the disease phenotype upon LXA_4_ treatment were summarized in Fig. 1; protocol 1. Parameters were pain, inflammation, oxidative stress, and histopathological alterations. Mice (n=6 per group per experiment) were stimulated in the right knee joint with an i.a. injection of TiO_2_ (3 mg/10 µl/ knee joint), as previously described (1). Twenty-four hours after (post-treatment) TiO_2_ stimulus, mice were treated with LXA_4_ (0.1, 1, or 10 ng) or vehicle (3.2% ethanol/saline) [100µl per animal, intraperitoneal (i.p.)]. Mechanical hyperalgesia and edema were evaluated twenty-four hours after TiO_2_ stimulus, before and after LXA_4_ treatment (1, 3, 5, 7, and twenty-four hours after LXA_4_ treatment on the first day and every other day from the 2nd to the 30th day). The dose and treatment time were chosen based on the results of mechanical hyperalgesia. After defining the most effective dose of LXA_4_ (10 ng/animal), the thermal hyperalgesia was evaluated every three days for 30 days. Articular edema was analyzed for 30 days, and knee joint lavages were collected on the 30th day for leukocyte recruitment analysis. The stomach was collected to determine toxicity after chronic treatment, on the 30th-day post-induction, assess myeloperoxidase (MPO) activity (stomach ulceration), and blood samples were used to assess serum levels of aspartate transaminase (AST), alanine transaminase (ALT) (liver damage), urea, and creatinine (renal damage). The knee joints were collected for histopathology analysis [hematoxylin-eosin stain (HE)].

The 2nd day after stimulus injection was chosen to elucidate the potential mechanisms of this lipid mediator in the early stages of TiO_2_-induced pain and inflammation (Fig. 1; protocol 2). The peak of inflammation was already achieved by the 2nd day, and higher leukocyte numbers comparing with the 30th day post-TiO_2_ injection. Therefore, on the 2nd day, the knee joint was used to determine leukocyte recruitment; cytokine levels as per enzyme-linked immunosorbent assay (ELISA) (TNF-α, IL-1β, IL-6, and IL-10); oxidative stress as per GSH and 2,2-azino-bis(3-ethylbenzothiazoline-6-sulfonate) (ABTS) measurement and Nrf2 mRNA expression by reverse transcriptase-quantitative real-time polymerase chain reaction (RT-qPCR). Synovial fluid leukocytes were also collected for p-NFκB and Nrf2 staining by immunofluorescence; and total ROS using the probe 2’,7’–dichlorofluorescein diacetate (DCF-DA) assay. Ipsilateral dorsal root ganglia (DRG) (corresponding to L4-L6 segments) were also dissected 2 days after TiO_2_ to determine calcium-fluo-4 imaging using confocal microscopy: transient receptor potential cation channel subfamily V member 1 (TRPV1) mRNA expression by RT-qPCR and TRPV1, transient receptor potential ankyrin 1 (TRPA1), ALX/FPR2 receptor, and p-NFκB staining by immunofluorescence.

The limited number of cells in the synovial fluid led us to use a peritonitis model induced by TiO_2_ to assess the cellular profile of recruited leukocytes and NF-κB activation in macrophages as well as if the treatment with LXA_4_ could modulate the responses triggered by TiO_2_ applying flow cytometry (Fig. 1; protocol 3). For this, mice received the intraperitoneal injection of TiO_2_ (30 mg/500 µl), and after twenty-four hours (post-treatment) they were treated with LXA_4_ (10 ng) or vehicle (saline) (100µl per animal, i.p.). After twenty-four hours, peritoneal washes were collected in FACS buffer (10mL per animal), and the cells were used to count total leukocyte recruitment, and flow cytometry for CD45, CD4, F4/80 and p-NFκB. All experimental conditions were standardized by our laboratory as previously published (1–4) and in preliminary experiments performed for this manuscript. The investigators were blinded to the treatment groups.

## Drugs, Reagents, and Antibodies

Materials were obtained from the following sources: saline solution (NaCl 0.9%; Frenesius Kabi Brasil Ltda, Aquiraz, CE, Brazil), LXA_4_, ≥ 95% purity, was purchased from Cayman Chemical (Ann Arbor, MI, USA) and pure TiO_2_, MW 79.90, was purchased from Synth (Diadema, SP, Brazil). ELISA kits for measurement of TNF-α, IL-1β, IL-6, and IL-10 were from eBioscience (Thermo Fisher Scientific, VIE, Austria). DCF-DA was purchased from Sigma-Aldrich (#D6883; San Luis, MO, EUA). Hank's Balanced Salt Solution (HBSS) was from Thermo Fisher Scientific (Waltham, MA, USA). RPMI medium was from (Thermo Fisher Scientific, VIE, Austria). The fluorescent antibodies were: anti-phosphorylated NF-κB p65 (#sc-136548; Mouse, Santa Cruz Biotechnology, Dallas, TX, USA); anti-Nrf2 (#sc-722; Rabbit, Santa Cruz Biotechnology, Dallas, TX, USA); FcR Blocking Reagent (Miltenyi Biotec, Cambridge, MA, USA); Ghost Dye™ Red 780 (Tombo, San Diego, CA, USA); PE anti-mouse CD45 antibody (#103106; Rat, BioLegend, San Diego, CA, USA); FITC anti-mouse CD45 antibody (103107; Rat, BioLegend, San Diego, CA, USA); FITC anti-mouse CD4 (#100406; Rat, BioLegend, San Diego, CA, USA); PerCP anti-mouse F4/80 antibody (#123126; Rat, BioLegend, San Diego, CA, USA); anti-capsaicin receptor antibody (#ab5566; Guinea pig, Merck Millipore, Burlington, MA, USA); anti-FPRL1/FPR2 antibody (#NLS1878ss; Rabbit, Novus biologicals, Englewood, CO, USA); anti-TRPA1/TSA antibody (#ab58844; Rabbit, Abcam, Cambridge, MA, USA); anti-mouse secondary antibody (Alexa Fluor 647-Goat, #115-605-003; Jackson ImmunoResearch, West Grove, PA, USA) and (Alexa Fluor 488-Goat anti-mouse; #A11001, Thermo Fisher Scientific, Waltham, MA, USA); anti-rabbit secondary antibody (Alexa fluor 488-Goat, #A-11008; Thermo Fisher Scientific, Waltham, MA, USA); anti-rabbit secondary antibody (Alexa fluor 647- Goat, #A32733; Thermo Fisher Scientific, Waltham, MA, USA); anti-Guinea pig secondary antibody (Alexa Fluor 488- Goat, #A11073, Thermo Fisher Scientific, Waltham, MA, USA). Hoechst 33342, trihydrochloride trihydrate was from Thermo Fisher Scientific (Waltham, MA, USA). 4′,6-Diamidine-2′-phenylindole dihydrochloride (DAPI) was from Thermo Fisher Scientific (Waltham, MA, USA). The panoptic kit for differential counts of recruited leukocytes was from Laborclin (Pinhais, PR, Brazil). Neurobasal-A medium (NBM) was purchased from Life Technologies (Thermo Fisher Scientific); Dispase II was from RocheApplied Sciences (Indianapolis, IN, USA); 4-(2-hydroxyethyl)-1- piperazine ethane sulfonic acid (HEPES)-buffered saline was from Millipore Sigma (Burlington, MA, USA); and Fluo‐4 a.m. was from Invitrogen (#F14201, Carlsbad, CA, USA).

## Evaluation of articular mechanical hyperalgesia

The knee joint mechanical hyperalgesia was evaluated. Mice were allowed to habituate to the apparatus for at least one h during three consecutive days before the measurements. Animals are placed in acrylic cages with a wire grid floor, and the stimulation was performed when the animals were quiet and with the four paws on the grid floor. This method consists of an electronic pressure meter with a force transducer fitted with a polypropylene tip (Electronic von Frey aesthesiometer; Insight instruments, Ribeirao Preto, SP, Brazil). We used a large tip (4.15mm2) to evaluate knee joint pain to exclude the subcutaneous effect (5). An increased perpendicular force was applied to the central area of the plantar surface to induce flexion of the tibiofemoral joint, followed by hind paw withdrawal. A digital aesthesiometer recorded the maximal intensity of the force applied [in grams (g)] when the paw was withdrawn. The test was performed in the time points of 1, 3, 5, 7, and twenty-four hours on the first day after LXA_4_ treatment and every other day from the 2nd to the 30th. The results were expressed as the mechanical withdrawal threshold in g.

## Evaluation of articular thermal hyperalgesia

For the heat hyperalgesia test, mice were allowed to habituate to the apparatus for at least two hours during three consecutive days before the measurements. After habituation, a baseline measurement was obtained. To measure pain sensitivity to a heat stimulus (heat hyperalgesia), we placed mice on a glass plate of a Hargreaves apparatus (Model 390G, IITC Life Science, Woodland Hills, CA, USA). A radiant heat source was used to stimulate the paw by gradually increasing the temperature of the plantar surface. The test was performed after LXA_4_ treatment and every three days until the 30th. The pain threshold was determined as the latency (in seconds) to evoke a response of paw withdrawal: paw flinches or licking. In this experiment, the device was set to 30% radiant heat source intensity and a cut-off time of 15s of exposure to prevent tissue damage.

## Articular edema measurements

Articular edema of the tibiofemoral joint was assessed through measurements of the transverse diameters using a caliper (Digmatic Caliper, Mitutoyo Corporation, Kanagawa, Japan). The edema was determined for each mouse knee joint by the difference indicated times post-stimulus and zero time. The test was performed at the time points of 1, 3, 5, 7, and twenty-four hours on the first day after LXA_4_ treatment and every other day from the 2nd to the 30th. The results were expressed as Δ mm/joint.

## Leukocyte migration

The total and differential counts of recruited leukocytes to the knee joint cavity were determined on the 2nd, and 30th day, as previously described (6). Briefly, knee joint cavities were washed with saline containing EDTA (50μl of solution/ 3 washes of 3.33μl), which was recovered to evaluate total and differential cell counts. In the peritonitis model, we collected the peritoneal washes (10mL of FACS buffer) on the 2^nd^ day after stimulus to evaluate leukocyte counts. Total cell counts were performed in the Neubauer chamber using Turk’s solution, and differential cell counts (100 cells per slide) were performed in slices stained with the panoptic kit under a light microscope (Olympus CX31RTSF, Tokyo, Japan). Results were expressed as total leukocytes, polymorphonuclear cells, and mononuclear cells (cells × 10^3^/ synovial cavity and cells × 10^6^/ peritoneal cavity).

## Liver and kidney toxicity assay

Blood samples were collected on the 30^th^ day post-TiO_2_ stimulus, centrifuged (0.4 rcf, 20 min, 4°C), and the serum was separated to assess the safety of treatment with LXA_4_. AST and ALT were used as markers of hepatotoxicity, and acetaminophen was used as a positive drug control (650 mg/kg, i.p.), diluted in sterile saline once). Urea and creatinine levels were used to evaluate nephrotoxicity, and diclofenac was a positive drug control (200 mg/kg, orally, diluted in sterile saline, once (7). The samples were processed according to the manufacturer’s instructions (Labtest Diagnóstico S. A., Brazil). Results were presented as U/mL (AST and ALT) or mg/dL (urea and creatinine) of serum.

## MPO activity

On the 30^th^ day post-TiO_2_ injection, samples of the stomach were harvested in 50 mM K_2_HPO_4_ buffer (pH 6.0) containing 0.5% hexadecyl trimethylammonium bromide (HTAB) and kept at – 80 °C until use. Frozen samples were homogenized using a tissue turrax (Tissue-Tearor 985370, BioSpec Products, Bartlesville, OK, USA) and centrifuged (2 min, 16,000g, 4 °C), and the resulting supernatant was assayed using a spectrophotometer (Multiskan GO Microplate Spectrophotometer, Thermo Fisher Scientific, Vantaa, Finland) for MPO activity determination at 450 nm. Briefly, 15μL of the sample was mixed with 200μL of 50 mM phosphate buffer (pH 6.0) containing 0.167 mg/mL O-dianisidine dihydrochloride and 0.0005% hydrogen peroxide. The MPO activity of samples was compared to a standard curve of neutrophils. Indomethacin (2.5 mg/kg, i.p., diluted in tris/HCl buffer, for 7 days) was used as positive drug control for stomach damage (8). The results were presented as MPO activity (number of neutrophils × 10^6^/ mg of tissue).

## Histopathological analysis

Knee joints were collected on the 30th day and fixed in 10% buffered formaldehyde. After decalcification in 20% EDTA disodium salt solution (pH 7,4) for 48 hours, the samples were processed for paraffin embedding. The tibiofemoral joint tissues were cut (10 μm) and stained with HE. The sections were examined, blinded, and scored by a pathologist in light microscopy. The score was determined by summing of synovial hyperplasia, inflammatory infiltrate, and vascular proliferation score, as described previously (4) Briefly, the degrees of the following parameters were: (a) synovial hyperplasia (from 0 = no pannus formation, to 3 = most severe pannus formation); (b) inflammatory infiltrate (from 0 = no inflammation, to 3 = most severe inflammation; and (c) angiogenesis (from 0 = no vascular proliferation, to 3 = most severe proliferation). Vascular proliferation was considered the number of capillary blood vessels. The final score was determined by summating all three parameters (a–c), resulting in a score for each sample expressed as the mean of six samples accordingly to the groups.

## Cytokine measurement

Knee joint samples collected on the 2nd day were homogenized in 500μL of buffer containing protease inhibitors. Samples were centrifuged (3000 rpm × 15 min × 4°C). TNF-α, IL-1β, IL-6, and IL-10 levels were determined from the supernatant by ELISA. The results were expressed as pg of cytokine/mg of protein.

## Synovial fluid leukocytes immunofluorescence

Knee joint cavities were washed in the 2nd day with FACS buffer (PBS and 0.5% BSA) containing EDTA (2mM), which was recovered to immunofluorescence assay as previously described with modifications (9). Samples were fixed with 4% paraformaldehyde for 30 min on ice. Then, samples were centrifuged (10 min, 4°C, 300g) and incubated with blocking buffer (PBS, 0.3% Triton) with bovine serum albumin (BSA) 3% for 1h and incubated with primary antibodies [anti-p-NFκB p65 (1:200) or anti-Nrf2 (1:200)] overnight (4°C). On the following day, samples were washed and incubated with secondary antibodies conjugated with Alexa Fluor 647-Goat anti-mouse (1:500) or Alexa Fluor 647-Goat anti-rabbit (1:500), and DAPI (1:500) was used as a nucleus marker. The samples were resuspended in PBS, placed on slides, and let to dry out at room temperature overnight. Imaging was performed using a confocal microscope (Leica TCS SP8, Leica, Wetzlar, Germany) with a 63x objective (p-NFκB p65) and a 63x objective with a zoom-in of 1.5 (Nrf2). Images were processed using Leica EL6000 software (Leica, Wetzlar, Germany). The fluorescence intensity of p-NFκB p65 of the different groups was quantified in randomly selected fields and analyzed from the mean fluorescence measured with the LAS X software (Leica Microsystems). The percentage of positive cells per field of Nrf2 was quantitated manually considering the following equation: total number of Nrf2^+^ cells times 100, divided by the total number of DAPI^+^ cells. Results were expressed as fluorescence intensity (p-NFκB p65) and percentage of positive cells per field (Nrf2).

## Flow cytometry

Mice received i.p. injection of TiO_2_ (30 mg/500 µl), and twenty-four hours after (post-treatment), they were treated with LXA_4_ (10 ng) or vehicle (saline) (100µl per animal, i.p.). After twenty-four hours, peritoneal washes were collected in FACS buffer (10mL per animal) to perform flow cytometry analysis (10). Cells were centrifuged for 10 min at 300 *g*; the supernatant was discarded, and the pellet was resuspended in 200 μl of FACS buffer. The cell suspension was incubated on ice with mouse FcR Blocking Reagent for 10 min and then incubated for 30 min on ice with the following antibodies: anti-CD45-PE (1:200), anti-CD45-FITC (1:200), anti-F4/80-PerCP (1:200), anti-CD4 FITC (1:200) and ghost dye red (1:200). After the incubation, cells were fixed with 2% PFA for 30 minutes at room temperature followed by permeabilization with FACs with 0,5% de Triton and the intracellular staining with anti-p-NFκB p65 (1:200) overnight. On the following day, cells were washed and incubated with secondary antibody Alexa Fluor 488 Goat anti-Mouse (1:500) for 1h. Cells were centrifuged for 10 min at 300 *g*, and the pellet was resuspended in 200 μl of FACS 2% PFA. FACS assay was performed using Guava® easyCyte™. Data were analyzed and plotted using FCS express software. Results were expressed as cells × 10^6^/ cavity.

## Antioxidant capacity measurement

The knee joint samples collected on the 2nd day were processed as previously described (11–13) and the levels of synovial GSH were determined using a spectrophotometric method. Frozen samples of knee joints were homogenized in cold 0.02 M EDTA. The homogenates were treated with 50% trichloroacetic acid and centrifuged (15 min x 1,500 g). The resulting supernatants received 0.4 M Tris-HCl, pH 8.9; next, samples were vortex-mixed, and 10 mM dithiobis nitrobenzoic acid was added, followed by vortex-mixing. After these procedures, samples could stand for 5 minutes before being read at 412 nm. The standard curves were prepared using graded concentrations of GSH. The results were presented as nmols of GSH / mg of protein. The free-radical scavenging ability was determined using the ABTS assay (11,13). ABTS was diluted with phosphate buffer saline at pH 7.4 to an absorbance of 0.80 at 730 nm. Subsequently, 1.0 mL of diluted ABTS solution was mixed with 20μL of the supernatant. After 6 min, the absorbance was measured at 730 nm. The results were equated against a standard Trolox curve (1.5-30 μmol/L, final concentrations). The results are expressed as Trolox equivalents per milligram of protein.

## Total intracellular ROS detection

The DCF-DA fluorescent probe was used to determine the presence of ROS. Knee joint cavities were washed on the 2nd day with FACS buffer (PBS and 0.5% BSA) containing EDTA, which was recovered to DCF-DA assay. The recovered articular fluids were seeded on Nunc™ Glass Bottom Dishes for 30 min at 37°C. Samples were then loaded with 10 µM of DCF-DA for 30 min 37°C, washed with HBSS, and imaged in a Confocal Microscope (TCS SP8, Leica Microsystems) with a 63x objective. Total intracellular ROS detection was analyzed from the mean fluorescence measured with the LAS X software (Leica Microsystems).

## RT-qPCR

Total RNA was extracted from knee joints and DRGs (L4-L6) on the 2^nd^ day using the SV Total RNA Isolation System (Promega). The purity of total RNA was measured using a spectrophotometer (Multiskan GO Microplate Spectrophotometer, Thermo Fisher Scientific, Vantaa, Finland), and the wavelength absorption relationship (260/280) was between 1.8 and 2.0 for all preparations. Reverse transcription of total RNA to cDNA and qPCR was carried out using GoTaq® 2-Step RT-qPCR System (Promega) and specific primers. The qPCR reaction was performed in a StepOnePlus™ Real-Time PCR System (Applied Biosystems®). The relative gene expression was measured using the comparative 2−(ΔΔCq) method. Table S1 shows the primer sequences. The expressions of β-actin mRNA were used as the reference gene, and the results were expressed as mRNA expression (normalized to β-actin).

**Table S1.** Primer sequences for RT-qPCR

| **Gene** | **Sense** | **Antisense** |
| --- | --- | --- |
| *Nrf2* | 5´-TCACACGAGATGACGTTAGGGCAA-3´ | 5´-TACAGTTCTGGGCGGCGGACTTTAT-3´ |
| *Trpv1* | 5´-TTCCTGCAGAAGAGCAAGAAGC-3´ | 5´-CCCATTGTGCAGATTGAGCAT-3´ |
| *β-actin* | 5′-AGCTGC GTTTTACACCCTTT-3′ | 5′-AAGCCATGCCAATGTTGTCT-3′ |

## Calcium imaging experiments

DRGs samples (L4-L6) were collected on the 2^nd^ day, and calcium imaging was performed as previously described (2) DRGs were dissected into NBM, dissociated in 1 mg/ml collagenase A and 2,4 U/ml de dispase II in HEPES for 20 minutes at 37˚C. After trituration with decreasing size glass Pasteur pipettes, DRG cells were centrifuged over a 10% BSA gradient, plated on laminin‐coated cell culture dishes. DRGs were then loaded with 1.2 μM of Fluo‐4 a.m. in NBM, incubated for 30 min 37°C, washed with HBSS, and imaged in a Confocal Microscope (TCS SP8, Leica Microsystems). DRG plates were recorded for 6 min to evaluate TRPV1 and TRPA1 activation, which was divided into 2 min of initial reading (0-120s, baseline values), followed by stimulation with 100 nM capsaicin (a TRPV1 agonist, 120-240s) or 100 µM allyl isothiocyanate (AITC, a TRPA1 agonist, 120-240s), and 40 mM of KCl (240-360s), activates all neurons. Only the KCl-responsive cells were considered in the analyses of capsaicin-responsive or AITC-responsive cells. Calcium flux was analyzed from the mean fluorescence (KCl responsive neurons) measured with the LAS X software (Leica Microsystems).

## DRG immunofluorescence

For immunofluorescence, DRGs of L4–L6 segments from Swiss were collected on 2nd day and maintained in 4% paraformaldehyde (PFA, for twenty-four hours), then in 30% sucrose (twenty-four hours) and 30% sucrose + OCT (1:1) (twenty-four hours) before inclusion with Optimum cutting temperature reagent (Tissue-Tek 1, O.C.T. Compound, IA018, ProSciTech, Australia) and 10 μm sections were cut in a cryostat and processed for immunofluorescence. After that, the slides were incubated with blocking buffer (PBS, 0.3% Triton) with bovine serum albumin (BSA) 3% for 1h and incubated with primary antibodies anti-TRPV1 (1:500); anti-FPRL1/FPR2 antibody (ALX/FPR2 receptor; 1:200); anti-TRPA1/TSA (1:100); anti-p-NF-κB p65 (1:200)] overnight (4°C). On the following day, the slides were washed and incubated with secondary antibodies conjugated with Alexa Fluor 488- Goat Anti-Guinea pig (1:500); Alexa Fluor 647 Goat anti-Rabbit (1:500); Alexa Fluor 488 Goat anti-Rabbit (1:500); Alexa Fluor Goat anti-Mouse (1:500). Hoechst 33342, trihydrochloride trihydrate (1:500) or DAPI (1:500) were used for nuclear staining. Imaging was performed using a confocal microscope (Leica TCS SP8, Leica, Wetzlar, Germany) with a 20x objective with a zoom-in of 1.0. Images were processed using Leica EL6000 software (Leica, Wetzlar, Germany). The results are expressed as the number of positive cells per area or percent of positive cells (double stained) as per manual quantitation.

## Statistical analysis

Data were analyzed using GraphPad Prism statistical software (GraphPad Software, Inc., USA-500.288, version 8.0). The results were presented as means ± SEM for parametric data and medians and interquartile ranges for non-parametric data. To this end, we used Shapiro–Wilk normality test and Brown-Forsythe homogeneity tests. For in vivo experiments, n of 6, 8, or 10 mice in each group per experiment and represent two separate experiments depending on the methodology (indicated in the figure legends). *In vitro* experiments with DRG samples were performed using an n of 4 pools [DRGs (L4-L6) of 10 mice to form 1 pool] per group and represent two separate experiments. Two-way repeated-measures analysis of variance (ANOVA) followed by Tukey’s post-test was used to compare all groups and doses when responses were measured at different times after the stimulus injection. The analyzed factor were treatments, time, and time versus treatment interaction. Parametric results were evaluated by one-way ANOVA followed by Tukey’s post-test for data from a single time point. Kruskal–Wallis followed by Dunn post-test or two-way were used for non-parametric results. P<0.05 was considered significant.

1. **Supplementary Figures**


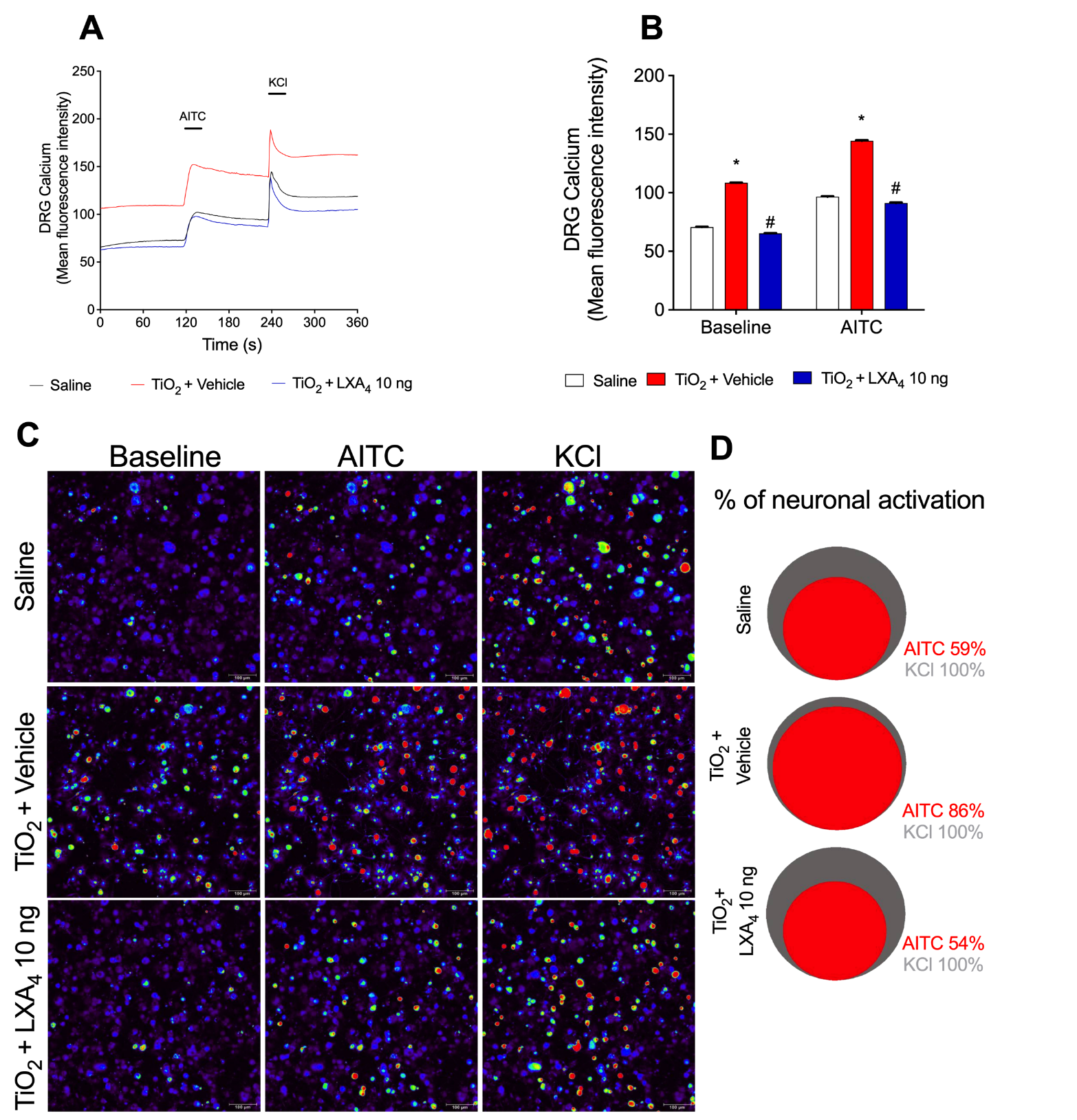


**Figure S1.** LXA_4_ reduces TiO_2_-induced TRPA1 activation on DRG neuron. Mice received a single treatment of LXA_4_ (10ng/ animal) starting 24h after i.a. injection of TiO_2_ (3mg/ joint), and on the 2nd day, the DRGs samples (L4-L6) were collected for calcium imaging using Fluo-4 a.m. probe. Panel **(A)** displays the traces of representative DRG fields of fluorescence of calcium-fluo-4 at baseline (0‐s mark) and that following the stimulus with AITC (a TRPA1 agonist, 120‐s mark), and KCl (activates all neurons, starting at the 240‐s mark). Panel **(B)** presents the mean fluorescence intensity of each group. Panel **(C)** shows representative fields of DRG neurons (baseline fluorescence, the fluorescence after AITC, and after KCl). Panel (**D**) shows Venn Diagram comparing the percentage of neurons that responded to AITC activation (red) within those responded to KCl stimulation (grey). Results are expressed as mean ± SEM, n = 4 DRG seeded plates (each plate is a neuronal culture pooled from 10 mice) per group per experiment, two independent experiments (*p<0.05 vs. saline group; #p<0.05 vs. TiO_2_ group, two-way ANOVA followed by Tukey’s post-test).


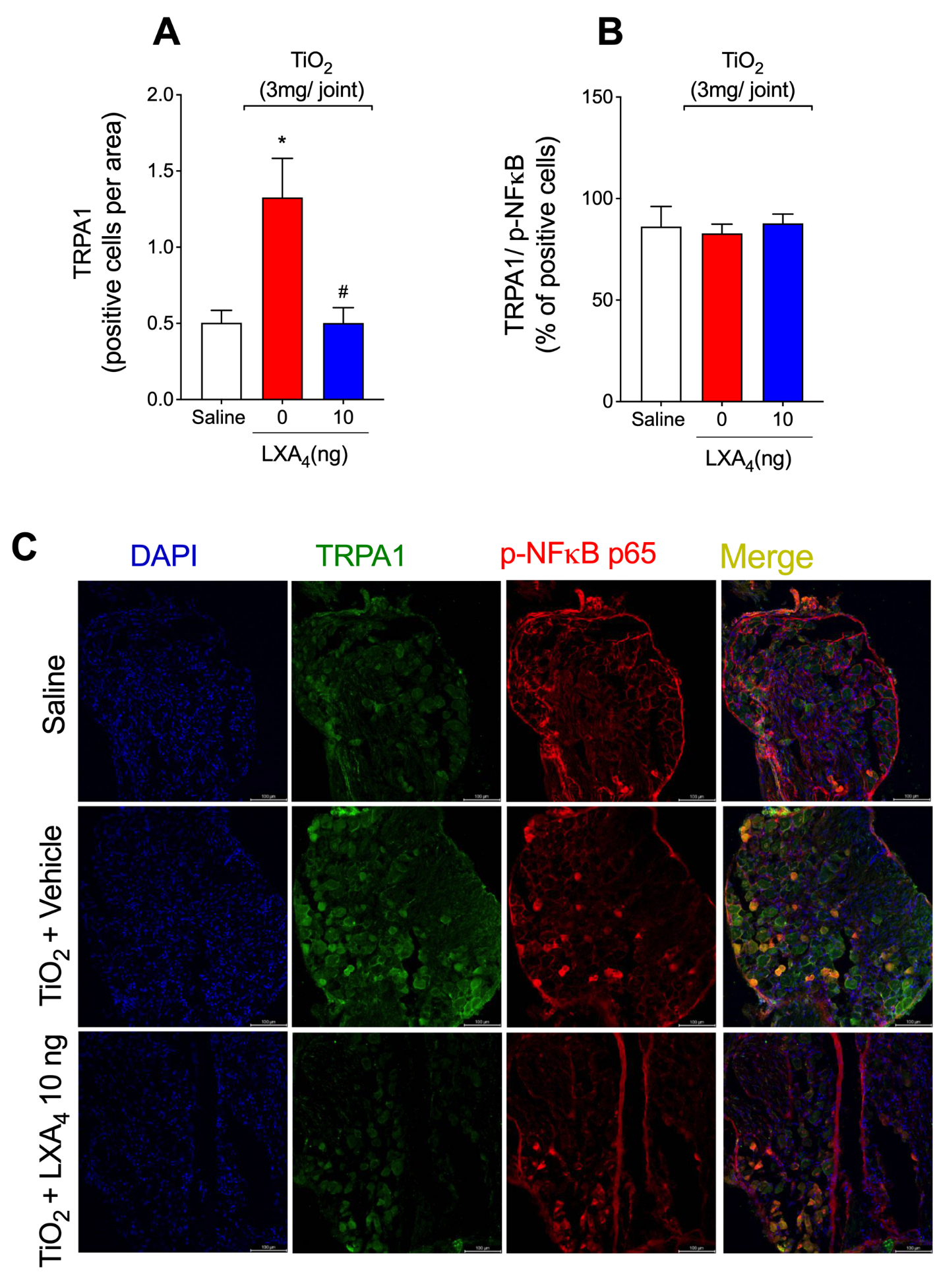


**Figure S2.** LXA_4_ inhibits NF-κB activation in TRPA1 positive neurons induced by TiO_2_. On the 2nd day of the model, DRGs samples (L4-L6) were dissected for TRPA1 and p65 p-NFκB staining by immunofluorescence technique. Panels **(A and B)** shows the quantitation analyses of the number of TRPA1 positive cells per area **(A)** and TRPA1 cells co-stained with phosphorylated p65 NF-κB (percent of positive cells) **(B)**. The panel **(C)** shows the representative images of TRPA1^+^ cells (green), p-NFκB^+^ cells (red) and merge of double labeling of TRPA1 and p-NFκB in DRG samples (20x magnification with 1.0 zoom in). The nuclear staining was performed by DAPI. Results are expressed as mean ± SEM, n=8 mice per group per experiment (*p<0.05 vs. saline group; #p<0.05 vs. TiO_2_ group, one-way ANOVA followed by Tukey’s post-test).

**References**

1. Borghi SM, Mizokami SS, Pinho-Ribeiro FA, Fattori V, Crespigio J, Clemente-Napimoga JT, Napimoga MH, Pitol DL, Issa JPM, Fukada SY, et al. The flavonoid quercetin inhibits titanium dioxide (TiO2)-induced chronic arthritis in mice. *J Nutr Biochem* (2018) 53:81–95. doi: 10.1016/j.jnutbio.2017.10.010

2. Fattori V, Pinho-Ribeiro FA, Staurengo-Ferrari L, Borghi SM, Rossaneis AC, Casagrande R, Verri Jr. WA. The specialised pro-resolving lipid mediator maresin 1 reduces inflammatory pain with a long-lasting analgesic effect. *Br J Pharmacol* (2019) 176:1728–1744. doi: 10.1111/bph.14647

3. Ferraz CR, Carvalho TT, Fattori V, Saraiva-Santos T, Pinho-Ribeiro FA, Borghi SM, Manchope MF, Zaninelli TH, Cunha TM, Casagrande R, et al. Jararhagin, a snake venom metalloproteinase, induces mechanical hyperalgesia in mice with the neuroinflammatory contribution of spinal cord microglia and astrocytes. *Int J Biol Macromol* (2021) 179:610–619. doi: 10.1016/j.ijbiomac.2021.02.178

4. Manchope MF, Artero NA, Fattori V, Mizokami SS, Pitol DL, Issa JPM, Fukada SY, Cunha TM, Alves-Filho JC, Cunha FQ, et al. Naringenin mitigates titanium dioxide (TiO2)-induced chronic arthritis in mice: role of oxidative stress, cytokines, and NFkappaB. *Inflamm Res* (2018) 67:997–1012. doi: 10.1007/s00011-018-1195-y

5. Guerrero ATG, Verri WA, Cunha TM, Silva TA, Rocha FAC, Ferreira SH, Cunha FQ, Parada CA. Hypernociception elicited by tibio-tarsal joint flexion in mice: A novel experimental arthritis model for pharmacological screening. *Pharmacol Biochem Behav* (2006) 84:244–251. doi: 10.1016/J.PBB.2006.05.008

6. Cobelli N, Scharf B, Crisi GM, Hardin J, Santambrogio L. Mediators of the inflammatory response to joint replacement devices. *Nat Rev Rheumatol* (2011) 7:600–608. doi: 10.1038/NRRHEUM.2011.128

7. Fattori V, Borghi SM, Guazelli CFS, Giroldo AC, Crespigio J, Bussmann AJC, Coelho-Silva L, Ludwig NG, Mazzuco TL, Casagrande R, et al. Vinpocetine reduces diclofenac-induced acute kidney injury through inhibition of oxidative stress, apoptosis, cytokine production, and NF-κB activation in mice. *Pharmacol Res* (2017) 120:10–22. doi: 10.1016/J.PHRS.2016.12.039

8. Wallace JL, McKnight GW, Bell CJ. Adaptation of rat gastric mucosa to aspirin requires mucosal contact. *Am J Physiol* (1995) 268: doi: 10.1152/AJPGI.1995.268.1.G134

9. Bussmann AJC, Borghi SM, Zaninelli TH, dos Santos TS, Guazelli CFS, Fattori V, Domiciano TP, Pinho-Ribeiro FA, Ruiz-Miyazawa KW, Casella AMB, et al. The citrus flavanone naringenin attenuates zymosan-induced mouse joint inflammation: induction of Nrf2 expression in recruited CD45+ hematopoietic cells. *Inflammopharmacology* (2019) 27:1229–1242. doi: 10.1007/S10787-018-00561-6

10. Fattori V, Pinho‐Ribeiro FA, Staurengo‐Ferrari L, Borghi SM, Rossaneis AC, Casagrande R, Verri WA. The specialised pro‐resolving lipid mediator maresin 1 reduces inflammatory pain with a long‐lasting analgesic effect. *Br J Pharmacol* (2019) 176:1728–1744. doi: 10.1111/bph.14647

11. Borghi SM, Carvalho TT, Staurengo-Ferrari L, Hohmann MS, Pinge-Filho P, Casagrande R, Verri Jr. WA. Vitexin inhibits inflammatory pain in mice by targeting TRPV1, oxidative stress, and cytokines. *J Nat Prod* (2013) 76:1141–1149. doi: 10.1021/np400222v

12. Casagrande R, Georgetti SR, Verri Jr. WA, Jabor JR, Santos AC, Fonseca MJ. Evaluation of functional stability of quercetin as a raw material and in different topical formulations by its antilipoperoxidative activity. *AAPS PharmSciTech* (2006) 7:E10. doi: 10.1208/pt070110

13. Hohmann MS, Cardoso RD, Pinho-Ribeiro FA, Crespigio J, Cunha TM, Alves-Filho JC, da Silva R v, Pinge-Filho P, Ferreira SH, Cunha FQ, et al. 5-lipoxygenase deficiency reduces acetaminophen-induced hepatotoxicity and lethality. *Biomed Res Int* (2013) 2013:627046. doi: 10.1155/2013/627046
